# Phylogeny and Historical Biogeography of Asian *Pterourus* Butterflies (Lepidoptera: Papilionidae): A Case of Intercontinental Dispersal from North America to East Asia

**DOI:** 10.1371/journal.pone.0140933

**Published:** 2015-10-20

**Authors:** Li-Wei Wu, Shen-Horn Yen, David C. Lees, Chih-Chien Lu, Ping-Shih Yang, Yu-Feng Hsu

**Affiliations:** 1 The Experimental Forest, College of Bio-Resources and Agriculture, National Taiwan University, Nantou, Taiwan; 2 Department of Biological Sciences, National Sun Yat-Sen University, Kaohsiung, Taiwan; 3 Department of Zoology, University of Cambridge, Cambridge, United Kingdom; 4 Department of Life Science, National Taiwan Normal University, Taipei, Taiwan; 5 Department and Graduate Institute of Entomology, National Taiwan University, Taipei, Taiwan; The University of Western Australia, AUSTRALIA

## Abstract

The phylogenetic status of the well-known Asian butterflies often known as *Agehana* (a species group, often treated as a genus or a subgenus, within *Papilio sensu lato*) has long remained unresolved. Only two species are included, and one of them especially, *Papilio maraho*, is not only rare but near-threatened, being monophagous on its vulnerable hostplant, *Sassafras randaiense* (Lauraceae). Although the natural history and population conservation of “*Agehana*” has received much attention, the biogeographic origin of this group still remains enigmatic. To clarify these two questions, a total of 86 species representatives within Papilionidae were sampled, and four genes (concatenated length 3842 bp) were used to reconstruct their phylogenetic relationships and historical scenarios. Surprisingly, “*Agehana*” fell within the American *Papilio* subgenus *Pterourus* and not as previously suggested, phylogenetically close to the Asian *Papilio* subgenus *Chilasa*. We therefore formally synonymize *Agehana* with *Pterourus*. Dating and biogeographic analysis allow us to infer an intercontinental dispersal of an American ancestor of Asian *Pterourus* in the early Miocene, which was coincident with historical paleo-land bridge connections, resulting in the present “East Asia-America” disjunction distribution. We emphasize that species exchange between East Asia and America seems to be a quite frequent occurrence in butterflies during the Oligocene to Miocene climatic optima.

## Introduction

Morphological and/or molecular data analyses have provided strong support for many monophyletic clades within the family Papilionidae. There is now wide consensus that Papilionidae is sister to all other Papilionoidea, which include Hesperiidae and Hedylidae [1–3]. In the past decade, based on substantial sequence data and quite comprehensive taxon sampling, knowledge of swallowtail butterfly phylogeny has become increasingly robust [4–10]. Recently, tribal relationships within the largest subfamily Papilioninae have been revised, including *Meandrusa* and *Teinopalpus* [10]. These two Asian genera, classically treated as members of the tribe Papilionini [11–13], are now placed in the tribe Teinopalpini [10].

The *Papilio elwesi*-species group [11] (as we refer to the most commonly used genus-group name *Agehana* Matsumura, 1936) has been considered an important lineage to include to clarify phylogenetic relationships within *Papilio sensu lato* [14]. This group is endemic to eastern Asia, and is morphologically very distinct from other Asian butterflies. Two species are recognized [15]: the continental species, *P*. *elwesi* Leech 1889, distributed from Southwest to Southeast mainland China, and the insular species, *P*. *maraho*, endemic to Taiwan. The latter species has the highest profile due to its rarity and listing as a vulnerable species in the IUCN Red Data Book [16], and it has been classified as an endangered species locally since 1994 [17]. Both species exhibit a fragmented distribution like their hosts, but *P*. *elwesi* has a larger population range than *P*. *maraho*. This is not surprising because immatures of the former can utilize both wider distributed Lauraceae (*Sassafras tzumu*) and Magnoliaceae (*Liriodendron chinense*, and *Magnolia officinalis*) [15], while the latter only feeds on an IUCN vulnerable plant, *S*. *randaiense*, sparsely distributed in sunny environments of forest gaps at medium elevations of the Central Mountain Range of Taiwan [18].

Presently *Papilio sensu lato* is divided into four major lineages [10, 14, 19]. The first is the subgenus *Heraclides* Hübner, 1819 (the *Heraclides*-clade), comprising 29 species and only occurs in the Americas. The second includes the subgenera *Pterourus* Scopoli, 1777 and *Chilasa* Moore, 1881 (the *Pterourus*-clade), which are respectively endemic to America (20 spp.) and Asia (13 spp.). The third is the largest lineage, consisting of *Papilio* Linnaeus, 1758 together with the subgenera *Princeps* Hübner, 1806, *Achillides* Hübner, (1819), *Druryia* Aurivillius, 1881, *Menelaides* Hübner, [1819], *Princeps* Hübner, (1807), *Sinoprinceps* Hancock, 1983, and *Eleppone* Hancock, 1979 (referred to hereafter as the *machaon*-clade, 143 spp.). The fourth lineage is the subgenus *Alexanoria* Koçak and Kemal, 2002, with just one species, *P*. *alexanor*. The phylogenetic position of the *elwesi*-species group is still ambiguous among different studies [11, 13, 19]. It has been treated as independent subgenus *Agehana* [13], or synonymized with the subgenus *Chilasa* [11].

Much effort has gone into assessing not only their classification and systematics, but also life cycle, ecology, and conservation evaluation of “*Agehana*” [13, 15, 20]. However, the precise placement of the *elwesi*-group has long remained enigmatic, despite a number of taxonomically quite densely sampled phylogenetic treatments of swallowtail butterflies in recent years, which did not however include this group [5, 14, 21]. Based on current evidence, the Old World danaine-mimetic subgenus *Chilasa* has been considered as the most plausible sister to this group [11, 13, 19]. The *elwesi*-group and *Chilasa* not only exhibit mimicry (the former have an *Atrophaneura*-like wing pattern), but also share a similar distribution [22]. However, the *elwesi*-group has very different larval and adult morphology to typical members of *Chilasa* [13]. Meanwhile, the specialized host-plant relationships of this *elwesi*-group could also shed light on its phylogenetic relationship, since both *Sassafras* spp. (Lauraceae) and *Liriodendron* spp. (Magnoliaceae), exhibit a disjunct distribution between Eastern Asia and North America [23], while only some *Pterourus* species (e.g. *P*. *glaucus* and *P*. *troilus*) have been recorded to use these two hostplant genera [24, 25]. The *elwesi*-species group has even been considered as a member of *Pterourus* in one work [26]. However, this phylogenetic hypothesis may be considered tentative only, because of no phylogenetic analyses on the *elwesi*-species group has ever been carried out with other *Pterourus*-clade members.

The possibility must then be considered that the *elwesi*-species group does not represent local evolution with Asian congeners. Recently, and not surprisingly, dispersal has been shown to be a major process shaping *Papilio* distributions [4, 21]. With increasing amount of molecular sequence data available for Papilionidae, the phylogenetic placement of the *elwesi*-group as well as the dispersal question can be objectively re-examined. Here, we take the opportunity to re-examine the relationships within the *Pterourus*-clade based on previous comprehensive phylogenetic analyses for the family Papilionidae [4, 14]. Then, we further revise the phylogenetic relationships of the *Pterourus*-clade and elucidate the historical biogeography of the *elwesi*-species group based on an evaluation of divergence times and the most probable historical scenario.

## Materials and Methods

### Sampling

This study adds sequences for new sampling of taxa both for the *elwesi*-group species (*P*. *elwesi* and *P*. *maraho*) and for seven related species, including six *Chilasa* members (*C*. *agestor*, *C*. *slateri*, *C*. *osmana*, *C*. *paradoxa*, *C*. *veiovis*, and *C*. *laglaizei*), and *Papilio bootes*. Other related species and outgroups were obtained using public domain sequences [2, 4, 8, 10, 14, 27] (species used in this study are listed in S1 Table). To evaluate the phylogenetic position of the *elwesi*-species group, a total of 42 species representatives were used (the 42-dataset; No. 1–42, S1 Table) based on previously reported phylogenetic relationships [4, 14]. In this 42-dataset, two species, *Battus philenor* and *Meandrusa sciron*, were set as functional outgroups [28] to investigate the phylogenetic position of the *elwesi*-group among the four major subdivision clades of *Papilio* [10, 14, 19]. Moreover, further taxa were added for calibrating dating points, this larger dataset is referred to as the 87-dataset, comprising 86 species including two specimens for the deep-branching species *Baronia brevicornis* (No. 1–87, S1 Table). This 87-dataset represents all butterfly families, including most major subfamilies and tribes of Papilionoidea for the dating estimation.

### Ethics Statement

None of our sampled materials involve vertebrates or cephalopods. *Papilio maraho* was the only species on the list of protected species in Taiwan, and we had permission for sampling this species that was issued by the Council of Agriculture (Taiwan). All the other species used in the manuscript are not listed as endangered species and were not collected from any locations that are national parks or natural reserves, thus no specific permission was required for sampling these species. All field studies performed in the present study did not involve endangered or protected species except for *P*. *maraho*.

### Molecular technologies

Genomic DNA was extracted from the thoracic muscle using the Purgene DNA Isolation kit (Gentra Systems, Minnesota, USA). Precipitated DNAs were resuspended in 50 μL of dH_2_O. The primers used for amplifying the mitochondrial cytochrome c oxidase 1 (*cox1*, 1530 bp), cytochrome c oxidase 2 (*cox2*, 681 bp), nuclear elongation factor 1 alpha (*Ef-1α*, 1225 bp) and *wingless* genes (*wg*, 403 bp) have been described previously [6, 15, 29–31], and novel primers which were designed for the *cox1*-*cox2* region of the *Papilio elwesi*-species group and for the partial *Ef-1α* gene of *Chilasa* members are listed in Table 1. Each polymerase chain reaction (PCR) was carried out in a final volume of 30 μL, with 0.2 μM of each primer. The following PCR settings were adopted: 2 min at 94°C, followed by 35 cycles of 30s at 94°C, 30s at 50–60°C, and 1–2 min at 72°C. The final elongation step was continued for 7 min at 72°C, and stopped at 4°C. If the above conditions failed, we amplified the fragments using a touchdown method: 2 min at 94°C, then following by 10 cycles of 30s at 94°C, 30s at 65°C decreasing 0.5°C degree each cycle, 1–2 min at 72°C, and then followed by 35 cycles of 30s at 94°C, 30s at 50°C, and 1–2.5 min at 72°C. The final elongation step was continued for 7 min at 72°C, and stopped at 4°C. The PCR products were run on 1.0% agarose gels in 1X TBE buffer to ensure that the lengths of PCR fragments were correctly amplified. DNA sequences were conducted using an ABI3730 DNA Analyzer (Applied Biosystems, Foster City, CA, USA).

**Table 1:** A list of novel primers used in the present study.

	Name	Sequence (5' to 3')
Mitochondrial primers for *cox1-cox2* region		
	C1-F1759	CATTTTGACTTTTACCCCCCTC
	C1-R2547	TGAAAATGAGCAACAACATAATA
Nuclear primers for *Ef-1α* gene		
	EF-548R	AACATGTTGTCTCCGTGCCA
	EF-608F	TGCCCTGGTTCAAGGGRTGGCA
	EF-969F	GACTCCAAGAACAACCCACCCA
	EF-1242R	ACRGTYTGTCTCATGTCACG

### Sequence treatment

Molecular sequences of *cox1*, *cox2*, *Ef*-*1α*, and *wg* genes were checked and assembled into contigs using Sequencher 4.8 (GeneCode, Boston, USA). Primer regions were cropped, and the uncompleted stop codons of *cox1* and *cox2* were removed to avoid length variation. The data sets were aligned according to amino sequence similarity by MUSCLE implied in MEGA5 [32]. Missing data and ambiguities were designated as IUPAC codes. All sequences were submitted to GenBank (GenBank accession numbers are listed in S1 Table; DNA alignment files can be obtained from S1 File). General sequence information was analyzed via the web server DIVEIN [33] and the results were double checked manually in Microsoft Excel. For phylogenetic analyses, aligned genes were concatenated via Microsoft Excel, and these datasets were converted to Fasta, Phylip, or Nexus format for further analyses.

### Phylogenetic analyses

A range of phylogenetic methods were used to infer phylogenies on the 42-dataset (S1 Table; S1 File). Maximum parsimony (MP) was performed in PAUP*10b [34]. Bayesian inference (BI) was carried out using MrBayes v. 3.2.1 [35], and Maximum Likelihood (ML) was performed in RAxML Pthreads-based version 7.0.4 [36, 37]. In the MP method, *Meandrusa sciron* and *Battus philenor* were set as outgroups and the remaining taxa as ingroups. The MP trees were reconstructed using heuristic searches, with starting trees determined by 1,000 random taxon additions, using the tree bisection-reconnection (TBR) branch swapping algorithm. All characters were treated as equally weighted. A strict consensus tree was computed in cases where multiple equally parsimonious trees were obtained. Bootstrap analyses were performed using heuristic searches, 100 random taxon additions, and the TBR algorithm. The number of bootstrap replicates was set to 1000.

Different data partitioning and substitution models would lead to varying branch lengths and consequently provide different phylogenetic topologies and dating estimates [38, 39]. To test our likelihood methods, we firstly divided our data matrix into six partitioning strategies (PSs). These are referred to as (1) no partition: four genes concatenated into one, (2) two partitions, one for mitochondrial genes and one for nuclear genes, (3) three partitions, one for the *cox1*+*cox2* genes, one for the *Ef-1a* gene, and one for the *wg* gene, (4) four partitions, for four genes, (5) nine partitions, for codon positions of *cox1*+*cox2*, *Ef-1α*, and *wg* genes, and (6) 12 partitions for codon positions of the four genes. The best-fit substitution model of each partition was evaluated by jModeltest 2 [40]. The 88 candidate models were set, and the corrected Akaike Information Criterion (AICc) was used to assign the optimal criterion (S2 Table). Moreover, we also used PartitionFinder 1.1.1 [41] to choose the best-fit substitution models and data partitions (hereafter referred to as best-fit PS). We divided our data matrix into 12 subsets (according to four genes and their codon positions) and relied on the PartitionFinder to estimate the best fit PS and substitution models. The result showed that there are eleven partitions and their best-fit substitution models are listed in S3 Table. To investigate which PS was the most strongly preferred [38], our seven PSs were all evaluated via the analysis of Bayes factors [42]. We took Bayes factors over ten as significantly different. Additional analysis details are described in our previous work [43].

In the BI method, a total of seven PSs were performed with eight chains (seven heated and one cold) and run for ten million generations and sampled trees every 100 generations. The convergence test of Marko Chain Monte Carlo (MCMC) chains was checked using Tracer 1.6 [44], and analyzed results (S2 File) were examined in AWTY [45]. When stationarity of MCMC processes was reached, the first 25% of sampled trees was discarded and the remaining trees were used for representing the posterior probability. For accessing high quality Bayesian phylogenies, the effective sample size (ESS) of each parameter was checked to ensure that all parameters were over 200. For both BI and ML methods, the single-outgroup setting was set to *Battus philenor*. In the ML method, seven partitioning strategies were set with the model of GTRGAMMA to each partition. All model parameters were estimated by RAxML. Nodal supports were evaluated using 1000 bootstrap replicates with 10 additional ML searches of each replicate.

### Estimation of divergence time

We took the 87-dataset and used relaxed Bayesian clock analysis to estimate divergence times using the software BEAST v1.8 [46]. The best-fit PS was found by PartitionFinder, which has eleven partitions divided by codon positions of each gene, except for the combined one including the third codon position of the *cox1* and *cox2* genes. The xml-file of this PS was created in the platform BEAUti, and used the following non-default settings: a relaxed clock using uncorrelated lognormal was selected; a speciation tree prior was set to Yule Process; the substitution model of each PS partition was set to best-fit models (S3 Table), and the prior parameters of “ucld.mean” were set to “lognormal” distributions. The analysis ran for 100 million generations, sampled every 1000 generations. Once the ESS values of parameters all exceeded 200, the first 10% of sampled trees was discarded and the remaining trees were used for dating purposes.

For molecular dating calibration, we used three butterfly fossils and set these calibration points following the rationale of Ho and Phillips [47]. The minimum age of divergence between Parnassiinae and Papilioninae was set to 48 Ma based on the fossil genus *Praepapilio* [48]. The minimum divergence boundary of Pierini was set to 34 Ma, based on the fossils of *Stolopsyche libytheoides* and *Oligodonta florissantensis*, which were recorded near the Eocene-Oligocene transition [49]. The third setting was the minimum divergence of *Vanessa* which was set to 34 Ma based on the fossil *Vanessa amerindica*, which was recorded at the Eocene-Oligocene boundary [50]. The maximum age of each fossil calibration point was set to 183 Ma based on the inferred origin of angiosperm diversification [51] although we note that this is likely to be an upper boundary as it predates the fossil history of flowering plants by at least 40 Ma.

Finally, to evaluate the effect of different PSs on the dating estimation, the PS1 to PS6 datasets were used for inferring dating schemes. The prior settings were set as the same as the best-fit PS, but substitution models of each PS were set to best estimate inferred by jModelTest (S2 Table).

### Biogeographical analysis

**Fig 1:**
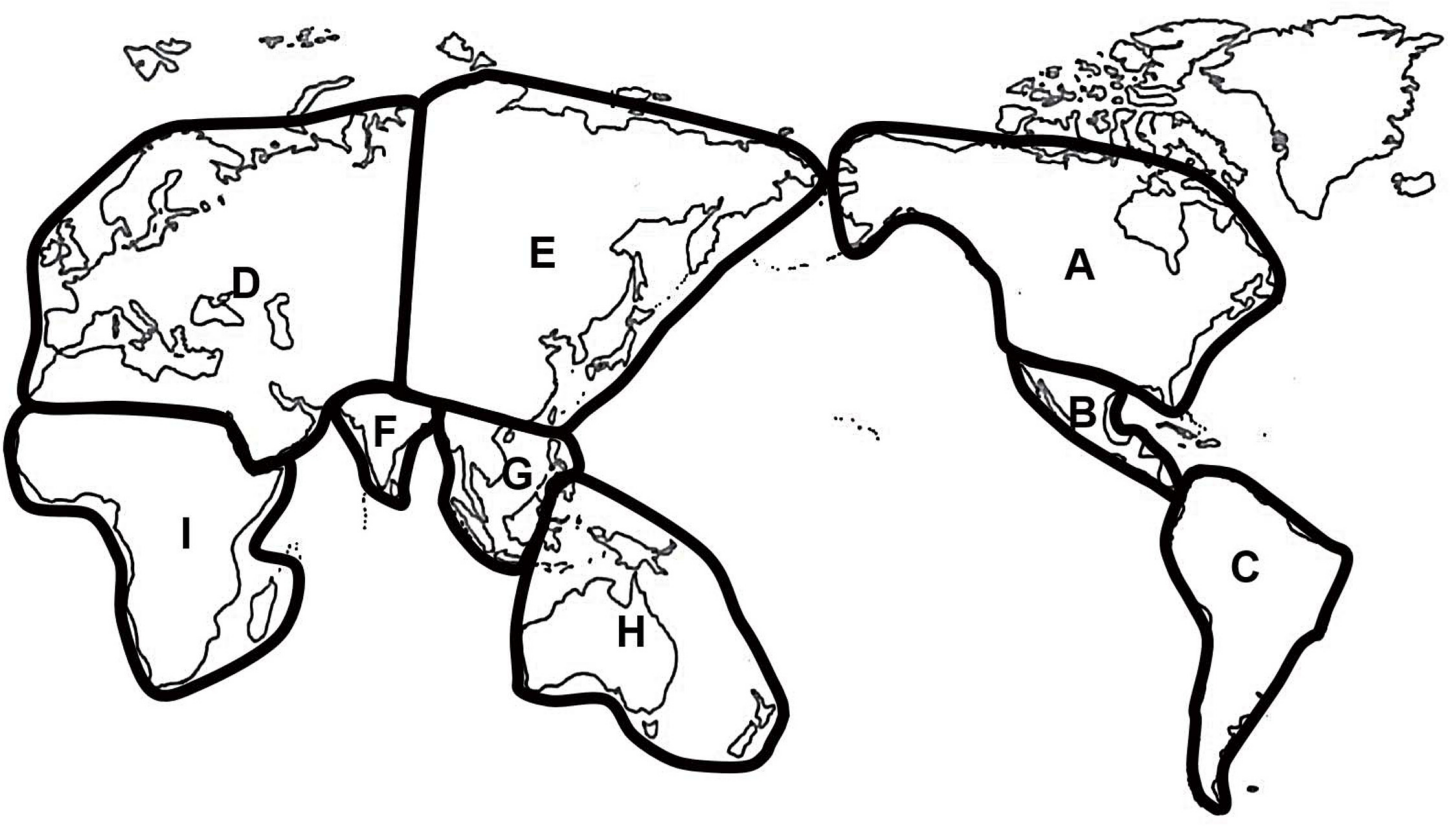
Defined Zoogeographic regions in this study. A: Nearctic region; B: Panamanian region; C: Neotropical region; D: West Palearctic and Saharo-Arabian regions; E: Sino-Japanese and East Palearctic regions; F: India; G: Oriental region; H: Oceanian and Australian regions; I: Afrotropical and Madagascan regions. The drawn map was modified from the time zone map of the World Factbook (https://www.cia.gov/library/publications).

Biogeographic reconstruction was performed using a dispersal-vicariance analysis [52] through a Bayesian MCMC process (Bayes-DIVA). This method can improve reliability because it provides clade posterior probabilities and phylogenetic uncertainties when multiple equally parsimonious reconstructions occur [39]. The current zoogeographic regions [53] were followed, but some regions were modified to suit our focal species (Fig 1): Afrotropical and Madagascan regions were combined into “I”; Oceanian and Australian regions were combined into “H”; Sino-Japanese and East Palearctic regions into “E”, whereas Saharo-Arabian and West Palearctic regions were combined into “D”. Nine areas were defined and showed in Fig 1. The consensus topology was inferred by the best-fit BI trees. The occurrence of each ancestral area was assigned a fractional ratio when multiple ancestral areas are present on a node [39]. We ran this analysis using the Reconstruct Ancestral State in Phylogenies software (RASP) [54]. With the non-default setting of one million MCMC generations, the temperature was set to 0.2, and the first 25% of trees was discarded. The model frequencies were set to “F81” and rate variation among sites was set to the Gamma distribution.

## Results

### Phylogenetic analyses

In the 42-dataset, the total alignment matrix contained 3842 bp (with 10% missing data), of which 1530 bp is from *cox1*, 684 bp from *cox2*, 1225 bp from *Ef-1α*, and 403 bp from *wg* gene. No stop codon was found throughout the whole dataset. A total of 1381 variable sites, with 1027 informative sites were found. In the 87-dataset (with 20% missing data), the alignment matrix and the aligned length of each gene are identical to the 42-dataset. The sum of total sampled lengths has 1755 variable sites, with 1495 informative sites.

**Fig 2:**
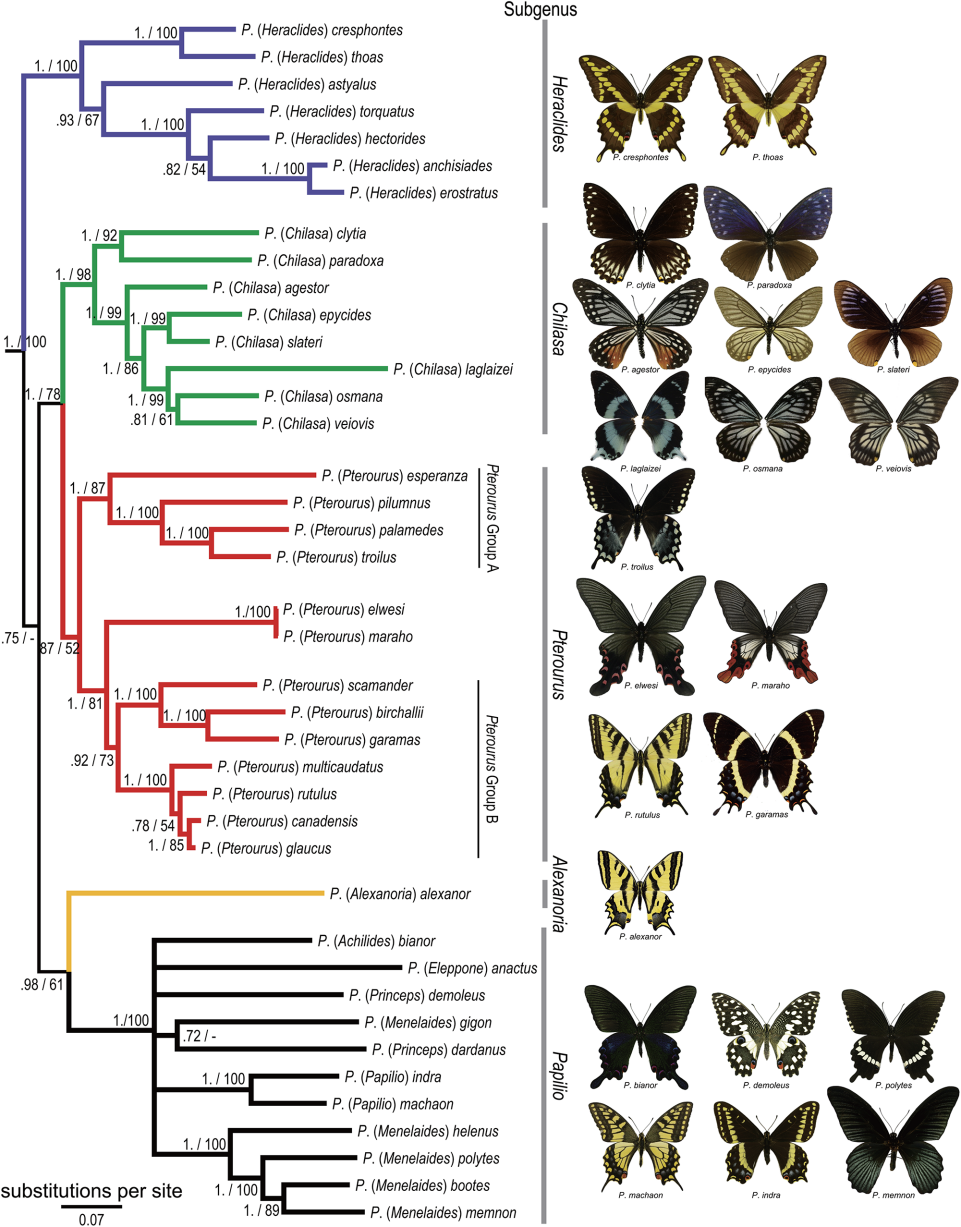
Phylogenetic relationships inferred by Bayesian best-fit PS and models. Bayesian posterior probabilities and ML bootstrap values are shown above the branches.

Phylogenetic relationships inferred by the best-fit BI and ML methods were congruent (Fig 2). Four major lineages were recognized, and the *Heraclides*-clade represents the sister lineage to the remaining three major clades. This four-clade relationship is also confirmed by other PS datasets (S3 File), except for the PS1 dataset, which presented the *machaon*-clade as sister to the other three clades. However, all the PS datasets showed that the relationships among the four major lineages had weak nodal supports. Focusing on the *Pterourus*-clade, the subgenus *Chilasa* and the *elwesi*-group grouped robustly within this clade, and the most intriguing finding was that inclusion of the *elwesi*-group within *Pterourus*, split this subgenus into two clades (*Pterourus* Group A and Group B; Fig 2). High support values showed that *Pterourus* Group B is more closely related to the *elwesi*-group than it is to *Pterourus* Group A. Moreover, the Asian *Chilasa* represents a sister clade to the remaining members in the *Pterourus*-clade.

In the MP method, the whole combined dataset resulted in one most equally parsimonious tree (S1 Fig, tree length = 5329 steps, CI = 0.358, RI = 0.439). The relationships of four major clades represented in the MP topology are mostly concordant with the results inferred by BI and ML methods. A significantly different relationship inferred by MP method is that the *elwesi*-group grouped with *Pterourus* Group B, whereas *Pterourus* Group A was grouped with *Chilasa*. However, the support values on those relationships were low.

### Estimation of divergence time

**Fig 3:**
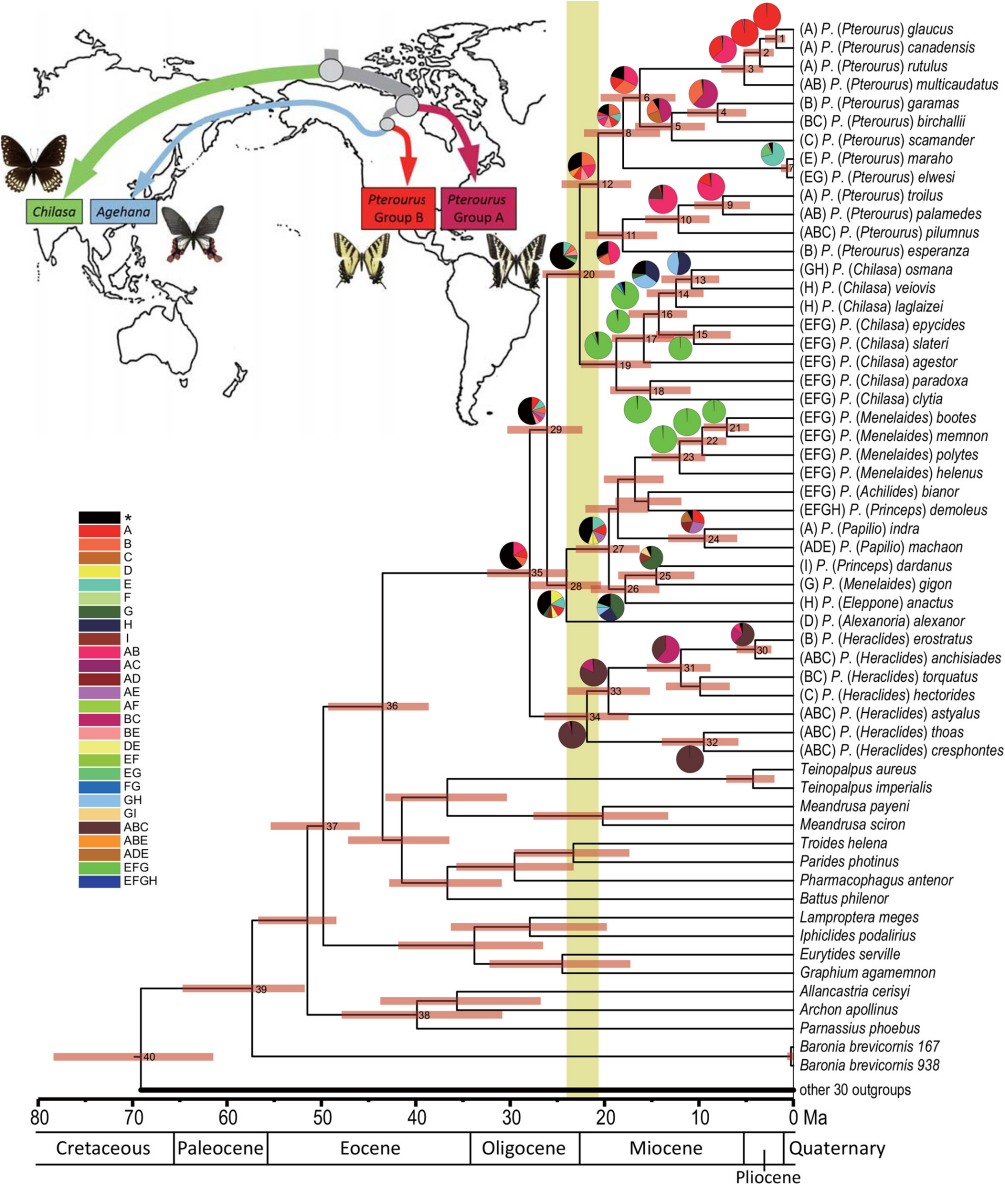
Calibration times and Bayes-DIVA results on inferred Bayesian consensus tree. Horizontal bars at nodes are 95% confidence intervals of estimated times. Regional codes are shown in the map, and the major fractional values from Bayes-DIVA are shown near the codes. Light brown bar: “Late Oligocene Climatic Optimum”.

The BEAST result inferred by the PS5 dataset has a high enough ESS value, and thus we take PS5 instead of the best-fit PS dataset to interpret our dating estimation (also see Bayes factor below). Our estimation of divergence times and their credibility intervals is shown in Fig 3 and S4 Table). The root of the tree was estimated at 69 Ma (node 40), whereas Papilionidae began to diversify at around 57 Ma (node 39). Focusing on the *Pterourus*-clade, this began to diverge as separated endemic lineages to Asia (*Chilasa*) and to America (*Pterourus* Group A and B) near the Oligocene-Miocene transition, approximately 23 Ma (node 20), a little before the divergence between the *elwesi*-group and *Pterourus* group B (Fig 2), circa 18 Ma (node 8).

In our BEAST analysis, we found that using many parameters in two PSs, the best-fit PS and PS6, we could not recover a high enough ESS value (below 100). Even when we increased the number of generations or turned off these low-ESS parameters, the ESS values resulting from these priors were still low. These outcomes might be caused by an incomplete data matrix (20% missing data, some gene sequences were not amplified; S1 Table), or by more partitioning strategies that would acquire smaller datasets to evaluate their parameters.

### Dispersal-Vicariance analysis

Focusing on the *Pterourus*-clade, the Bayes-DIVA outcomes (Fig 3 and S5 Table) postulates that the ancestor of the *elwesi*-group diversified in the East Asia (node 7, the fraction of E: 0.71, and EG: 0.20) and the most possible origin of the *elwesi*-group is in America with probabilities over 0.45 (node 8, A: 0.12, B: 0.22, and AB: 0.11). On the contrary, the ancestor of *Chilasa* diversified in the region spanning the Oriental region to India (node 19, FG: 0.91 and G: 0.03). Although its origin is still unclear (node 20, Fig 3), the diversification shows a dispersal trend from the region “EFG” towards the proximate Australian regions (GH and H) while that of the subgenus *Pterourus* (Group A and B) trends in the direction from Panamanian (B) to Nearctic (A) region (Fig 1).

### Bayes factors

In the 42-datasets for BI phylogenetic reconstruction, Bayes factors for our seven different PSs are shown in Table 2. These comparisons show that more partitioned datasets are strongly preferred over less partitioned ones. One exception is that best-fit PS (11 partitions) and PS5 (9 partitions) performed significantly better than PS6 (12 partitions). The former two PSs are also the most preferred strategies, whereas the combined datasets (PS1) comprise the least preferred one. In the 87-datasets for BEAST analysis, Bayes factors show that the dating scheme inferred by PS5 is most preferred strategy for our dataset, whereas the combined dataset (PS1) produced a significantly worse one (Table 3).

**Table 2:** Bayes factor comparisons between PSs of the 42-dataset. The rank of preference in PS is “best-fit = PS5 > PS6 > PS4 = PS3 = PS2 > PS1”. HM: harmonic mean; M1: model likelihood 1; M2: model likelihood 2.

M2 \ M1	HM	PS1	PS2	PS3	PS4	PS5	PS6
PS1	-29485.3						
PS2	-28936.5	12.62					
PS3	-28911.5	12.70	6.44				
PS4	-28901.8	12.74	7.09	4.54			
PS5	-27297.6	15.38	14.80	14.77	14.76		
PS6	-27546.6	15.14	14.47	14.44	14.42	-11.04	
best-fit PS	-27239	15.43	14.87	14.84	14.83	8.14	11.46

**Table 3:** Bayes factor comparisons between PSs of the 87-dataset. The rank of preference PS is “PS5 = best-fit PS>PS2 = PS3>PS4>PS1”. HM: harmonic mean; M1: model likelihood 1; M2: model likelihood 2.

M2 \ M1	HM	PS1	PS2	PS3	PS4	PS5
PS1	-62501.22					
PS2	-60944.94	14.70				
PS3	-60967.09	14.67	-6.20			
PS4	-61543.32	13.73	-12.79	-12.71		
PS5	-59748.06	15.84	14.17	14.21	14.99	
best-fit PS	-59771.82	15.82	14.13	14.17	14.96	-6.34

### Effect of different PSs on dating results

**Fig 4:**
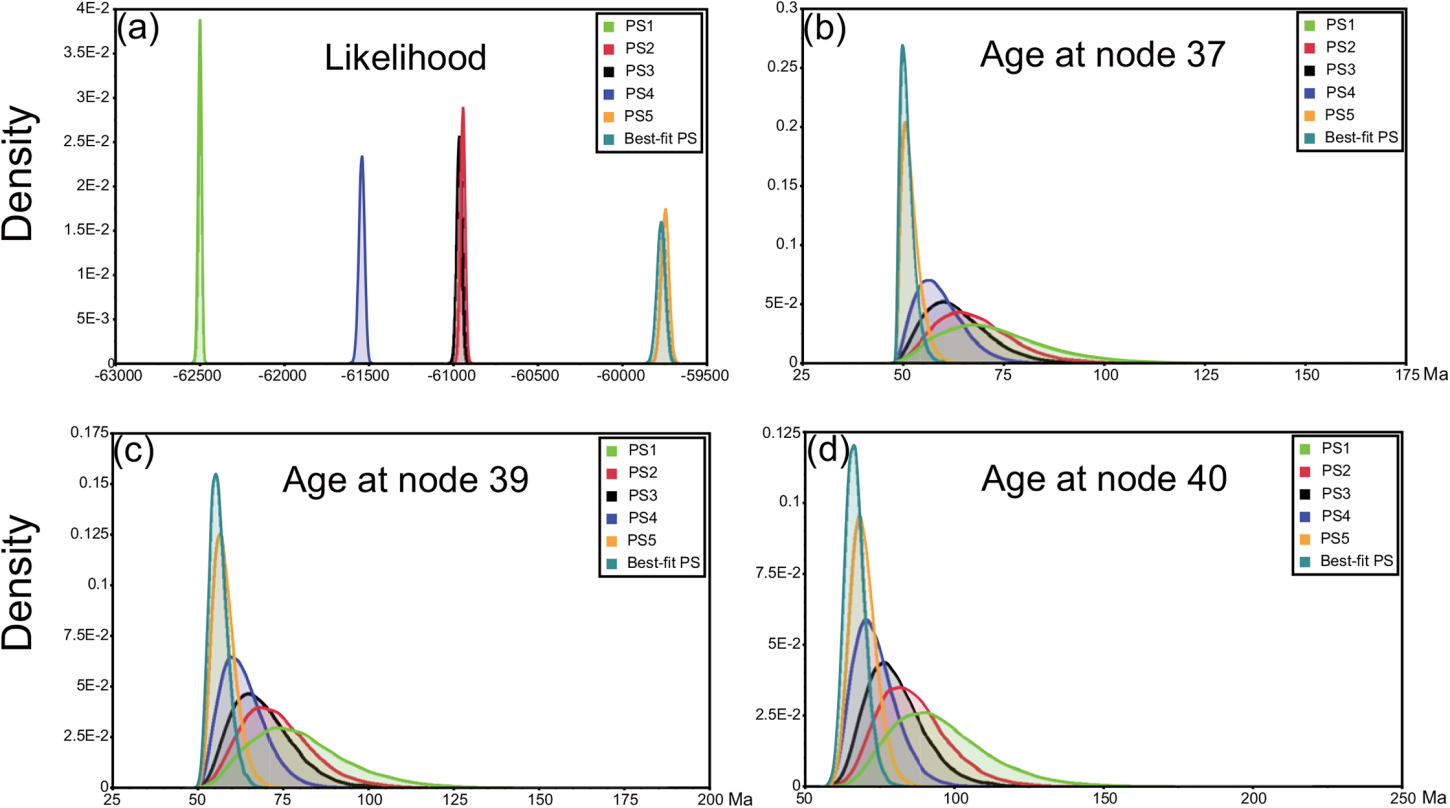
Comparisons of BEAST results among different partitioning strategies. (a): harmonic means; (b): estimated age at node 37; (c): estimated age at node 39; (d): estimated age at node 40.

Our results show that more partitioned PSs tend to obtain a younger dating scheme, and the range of 95% HPD would be narrower than for less partitioned strategies (Fig 4). The rooting point of Papilionoidea (node 40, Fig 3) is 69 Ma (95% HPD range 62–78 Ma) in PS5, but the values changed to 73 Ma (95% HPD range 61–88 Ma) in PS4, 80 Ma (95% HPD range 63–100 Ma) in PS3, 85 Ma (95% HPD range 65–110 Ma) in PS2, and 96 Ma (95% HPD range 67–128 Ma) in PS1. Similar tendencies are also found in other estimated dating points in Fig 4.

## Discussion

### Phylogenetic relationships

The monophyly of the large genus *Papilio sensu lato*, which comprises four major lineages, has been reported by Zakharov et al. [14] and is not in dispute. Our results are congruent with that study, but add more details for the *Pterourus*-clade. The most noteworthy finding is that the two Old World subgenera, *Chilasa* and *Agehana* (the *elwesi*-group), are both sister groups to the New World subgenus *Pterourus*, but that the placement of the *elwesi*-group causes the fission of *Pterourus* (Fig 2). This highly supported outcome suggests that the commonly used genus-group name, *Agehana*, should be synonymized with *Pterourus*. Another possible treatment, however, is to combine *Agehana* and *Pterourus* Group B within the subgenus *Pyrrhosticta* Butler, 1872 and to restrict the subgenus *Pterourus* to *Pterourus* Group A (Fig 2). We currently favor the former outcome as less disruptive to current subgeneric nomenclature, and we go as far as to propose a formal synonymy that can be tested with future data. Note that only about half of the American *Pterourus* species have been sampled. *Papilio zagreus*, for example, has been considered to represent another species-group [11]. This lineage should be added to clarify the phylogenetic relationships within the subgenus *Pterourus* and to confirm whether our radical proposal here that *Papilio* (*Pterourus*) should encompass both the Americas and South-East Asia and that subgenus *Agehana* be sunk and referred to in future as the *Papilio* (*Pterourus*) *elwesi*-species group, is borne out. Here, we treat the *Pterourus*-clade as comprising two reciprocal monophyletic subgenera (*Pterourus* and *Chilasa*), and delineate several species-groups for further classification (Table 4). Moreover, although previous morphological studies which attempted to subdivide *Papilio sensu lato* seem inconclusive [11–13], we notice that the *elwesi*-species group has strikingly similar larval stages to American *Pterourus* Group B [55, 56], supporting our nomenclatural proposal. It seems unlikely that such similarity would have arrived by mimetic convergence in different biogeographic regions, due to different sets of predators, and an alternative scenario is that the generalist adaptive qualities (e.g. snake mimicry) of a common ancestor were sufficiently repellent to predators to have been retained. Especially within the *P*. *homerus*-species group (Table 4), many species have double eye-spots on the prothorax and an “X”-shaped marking on the dorsal abdomen with blue spots (S2 Fig). In addition, the *elwesi*-group and their relatives have unusual hostplant associations (Lauraceae and Magnoliaceae) different from other *Papilio* swallowtails, which principally feed on Rutaceae [24, 25].

**Table 4:** Proposed species-group arrangement for the *Pterourus*-clade.

Species group	Name
**Subgenus *Chilasa***	
*agestor*-species group	*P*. *(Chilasa) agestor*
*clytia*-species group	*P*. *(Chilasa) clytia*
	*P*. *(Chilasa) paradoxa*
*laglaizei*-species group	*P*. *(Chilasa) laglaizei*
	*P*. *(Chilasa) moerneri*
	*P*. *(Chilasa) toboroi*
*slateri*-species group	*P*. *(Chilasa) epycides*
	*P*. *(Chilasa) slateri*
*veiovis*-species group	*P*. *(Chilasa) osmana*
	*P*. *(Chilasa) veiovis*
	*P*. *(Chilasa) carolinensis*
**Subgenus *Pterourus***	
*elwesi*-species group	*P*. *(Pterourus) elwesi*
	*P*. *(Pterourus) maraho*
*glaucus*-species group	*P*. *(Pterourus) appalachiensis*
	*P*. *(Pterourus) canadensis*
	*P*. *(Pterourus) eurymedon*
	*P*. *(Pterourus) glaucus*
	*P*. *(Pterourus) rutulus*
	*P*. *(Pterourus) multicaudatus*
*homerus*-species group	*P*. *(Pterourus) birchallii*
	*P*. *(Pterourus) garamas*
	*P*. *(Pterourus) hellanichus*
	*P*. *(Pterourus) homerus*
	*P*. *(Pterourus) menatius*
	*P*. *(Pterourus) scamander*
	*P*. *(Pterourus) warscewiczii*
	*P*. *(Pterourus) xanthopleura*
*troilus*-species group	*P*. *(Pterourus) esperanza*
	*P*. *(Pterourus) palamedes*
	*P*. *(Pterourus) pilumnus*
	*P*. *(Pterourus) troilus*
*zagreus*-species group	*P*. *(Pterourus) cacicus*
	*P*. *(Pterourus) euterpinus*
	*P*. *(Pterourus) zagreus*

### Historical biogeography of the *Pterourus*-clade

Our phylogeny of the *Pterourus*-clade illuminates biogeographic events between East Asia and America. A previous study hypothesized that two Old World lineages of this clade, *Chilasa* and *Alexanoria*, diverged from their New World relatives (*Pterourus*) around 24 Ma [21], and that their current disjunct distribution is a result of dispersal through the Bering land-bridge (BLB), following climate cooling events. Our results support this scenario, but we find that ensuing warming periods also provide gateways [57] that allowed the common ancestor of the *elwesi*-species group to disperse intercontinentally. In our case, we infer the divergence and diversification of the ancestor of the *elwesi*-species group from its sister, the *Pterourus* Group B (Fig 2), to have started around 18 Ma (Fig 3), a date that coincides with the early Miocene climatic optimum. During this period, deciduous and evergreen mixed forest reached relatively high paleolatitudes [57], and zoofaunal exchange became possible via BLB [58]. Our results are consistent with a dispersal of the *elwesi*-group ancestor to Eurasia through BLB. The ensuing global-cooling event [59] would have reduced and fragmented the butterflies’ ranges, accounting for their current relict East Asia-America distribution. This novel phylogenetic relationship showing the *P*. *elwesi*-group to be internal to *Pterourus*, contradicts the assertion that *Pterourus* butterflies only became isolated and diversified in Nearctic regions [21].

In contrast to the *elwesi*-species group, the diversification of *Chilasa* happened earlier, as we infer around 23 Ma (Fig 3 and S4 Table). The biogeographic scenario for *Chilasa* is a dispersal event from an ancestor among their American relatives during the Oligocene/Miocene transition, a period of warming (i.e. the Late Oligocene Climatic Optimum). This result is concordant with the previous study [21]. The hypothesis that *Chilasa* was Gondwanan in origin and dispersed from Australia to Southeast and India [11, 26] is clearly refuted by our dating results as well as previous dating estimates for Papilionidae [4, 21]. Instead, our Bayes-DIVA analysis allows us to infer a directional tendency of the diversification from East or Southeast Asia to Australian regions.

The diversification of American *Pterourus* dates back to around 21 Ma (Fig 3) from our analysis, by which time the island arc of proto-Central America had formed [60], followed by the “Great American Biotic Interchange” [61, 62]. Our results show that the *Pterourus* group formerly supposed to be endemic to the Americas comprises at least two monophyletic subunits (Fig 2). The diversification of Group A (14–22 Ma) shows a slight northward direction (Fig 3), and one lineage of group B (*P*. *multicaudata*, *P*. *rutulus*, *P*. *canadensis*, and *P*. *glaucus*) shows a pattern of diversification northward to North America (A). However, our sampling being incomplete for American taxa, we interpret these inferred directions and times of diversification with caution.

### Hostplant associations

Many *Pterourus* clade members (e.g. *P*. *glaucus*, and *P*. *scamander*) are moderately polyphagous butterflies as for the *elwesi*-group [24, 55, 63, 64]. The major hostplants for these Asian *Pterourus* butterflies are *Sassafras* and *Liriodendron*, and recently the immatures of *P*. *elwesi* were found on *Magnolia officinalis* (Magnoliaceae) [15]. Interesting, these hostplants all show a similar disjunct distribution between East Asia and North America [23]. Most recent dating studies reveal splits in two of these plant genera estimated at around 14 Ma for *Sassafras* and *Liriodendron* [65, 66], and around 18.5 Ma for *Magnolia* [66]. Interestingly, this dating estimate is very close to the divergence between the *P*. *elwesi*-group and its American relatives Group B in the early Miocene (18 Ma). Therefore, based on currently known hostplant records, and from our phylogenetic topologies, the current hostplant relationships of the *Pterourus*-clade suggest that their most recent common ancestor was also polyphagous, and also that the ancestor of the *elwesi*-species group might have had a broader host repertoire in the past, even though the two descendant species are quite narrowly specialized.

### Species within the East Asia-America disjunct distribution

Geology and climate change are evidently two of the most important factors promoting plant diversification and intercontinental exchange. Plants seem to be among the best model organisms for studying biogeographic disjunction between East Asia and North America [23], but an increasing number of zoofaunal examples point to common patterns of intercontinental disjunction and underlying historical biogeography, and are also important for unraveling the interplay between regional biodiversity and evolutionary processes [4, 58]. Currently, two phytophagous insect groups, aphids [67] and leaf beetles [68] seem to show a congruent biogeographic pattern with their hosts, emphasizing the significance of host distribution for diversification of specialized phytophagous insects. In our case study, the phylogenetic relationships and asymmetric distribution of *Pterourus* butterflies reveals the importance of ancient dispersal processes affecting butterfly composition in East Asia. Different climate warming periods are likely to have permitted exchanges of butterflies between East Asia and America. Most of those cases suggest diversification in Asia and dispersal of a few members to America [69, 70]. The classic documented case until now is the dispersal to the New World of *Polyommatus* butterflies no less than five separate times during the Miocene-Pleistocene [69]. So far only a few cases like our study and that of the satyrid *Palaeonympha opalina* [71] reveal clearly ancient dispersal events in the Asia-wards direction during the Oligocene to Miocene climatic optimum, whereas Asian-American riodinids show a striking intercontinental dispersal case that began as a diversification in the Neotropics, with a dispersal to Asia (~74 Ma) and a back dispersal to the Neotropics in late Eocene [72], In general, data for butterflies are still too few to suggest any clear trend in the past directionality of biotic exchange.

## Conclusions

Historical biogeographic analysis in the *Pterourus*-clade butterflies highlights the phenomenon of intercontinental exchange of tropical or subtropical taxa during paleoclimatic warming periods. Our study shows that the current distribution of the *P*. *elwesi*-group (now Asian representatives of *Pterourus*) represents a case of intercontinental dispersal from America. From a taxonomic viewpoint, we suggest that the genus-group name *Agehana* should be synonymized with *Pterourus* based on strong molecular support for monophyly and in accordance with larval morphology. This new arrangement communicates well their intriguing biogeographic history.

## Supporting Information

S1 Fig The most parsimonious topology. Values at nodes are bootstrap values.
(EPS)

S2 Fig The larval appearances of *P*. *maraho* (the *elwesi*-species group) and *P*. *garamas* (the *P*. *homerus*-species group). (TIF)

S1 File Associated alignment datasets and tree files used in this study. These comprise the 42-dataset and the 87-dataset mentioned in the text and eight tree files which were generated from PS1-PS6 and best-fit.
(RAR)

S2 File Convergence output of seven PS datasets. (PDF)

S3 File Bayesian and Maximum Likelihood trees based on PS1-6. Values at nodes correspond to posterior probabilities or ML bootstrap.
(PDF)

S1 Table Species list used in this study. GenBank accession numbers of each gene are listed.
(XLSX)

S2 Table Substitution model of each partition. The models were inferred by jModelTest 2 and judged by AICc using a corrected version for small samples.
(XLSX)

S3 Table Substitution models of best-fit PS. The best-fit partitions and models were inferred by PartitionFinder using the AICc criterion.
(XLSX)

S4 Table Divergence times (Ma) with their 95% HPD intervals at nodes of Fig 3. (XLSX)

S5 Table The possible ancestral areas inferred by Bayes-DIVA at nodes of Fig 3. “*”: uncertainty fraction
(XLSX)
