# Supplementary figures and images for: Phylogeny and Historical Biogeography of Asian Pterourus Butterflies (Lepidoptera: Papilionidae): A Case of Intercontinental Dispersal from North America to East Asia
Source: PLoS One. 2015 Oct 20;10(10):e0140933. doi: 10.1371/journal.pone.0140933 (PMC4617649; doi:10.1371/journal.pone.0140933)

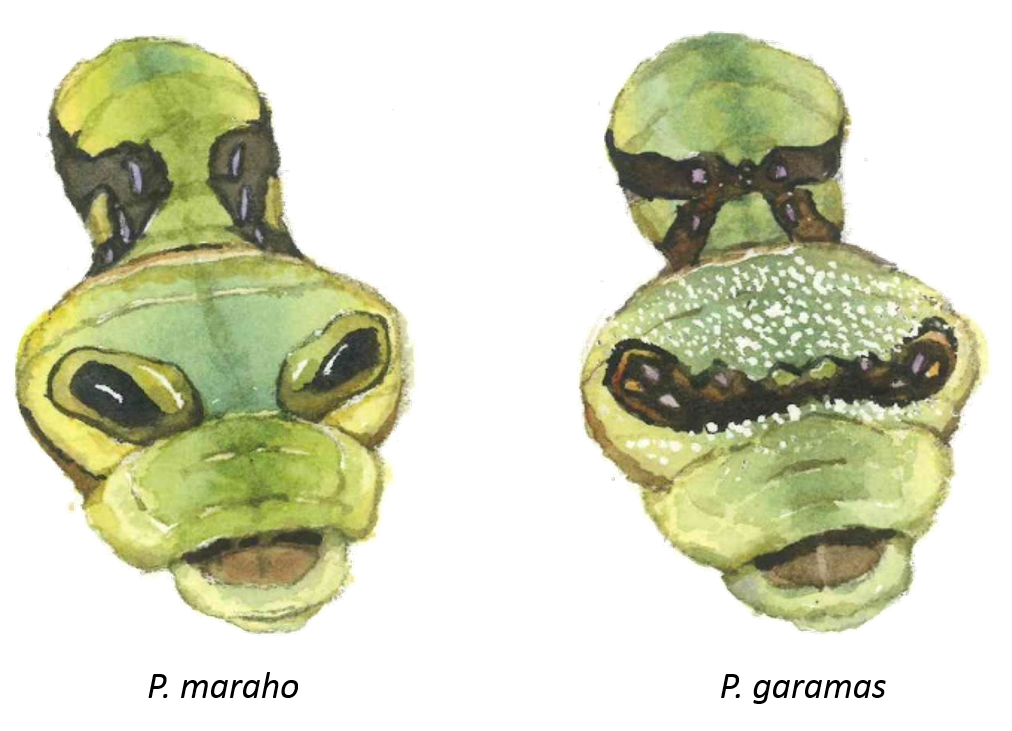

Supplement: S2 Fig — (TIF) [file pone.0140933.s002.tif]

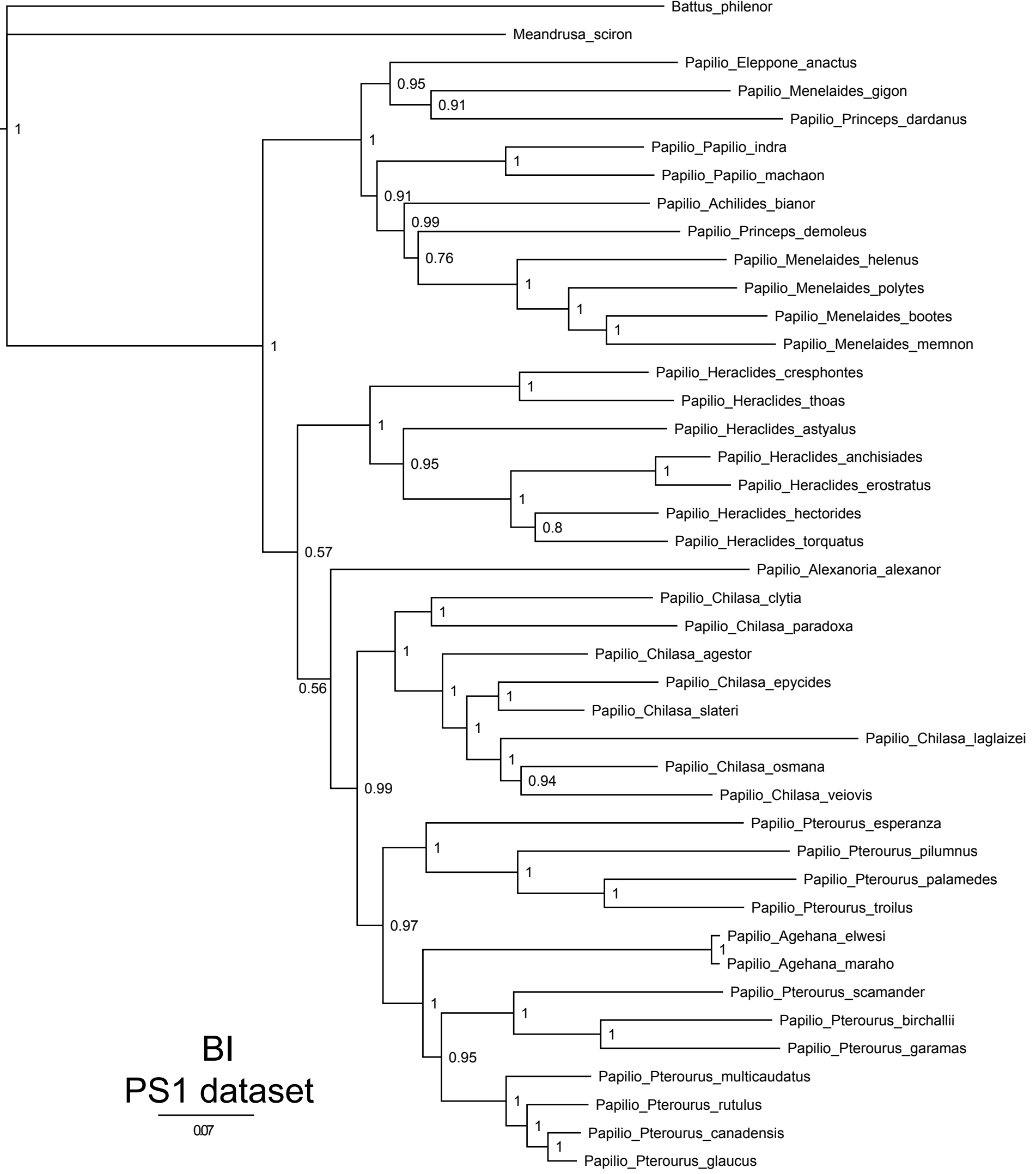

BI  
PS1 dataset  
0.07

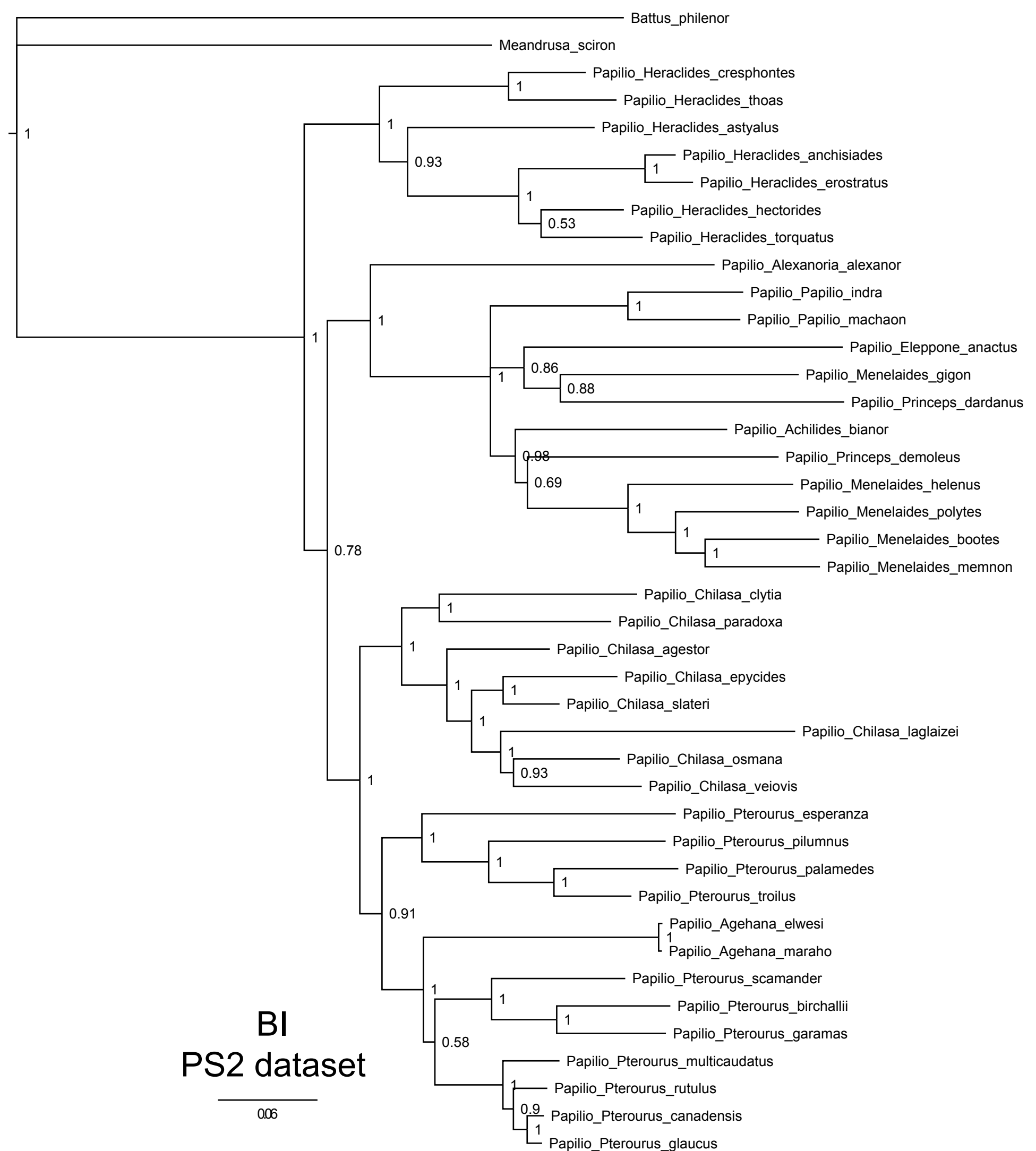

BI  
PS3 dataset  
0.05

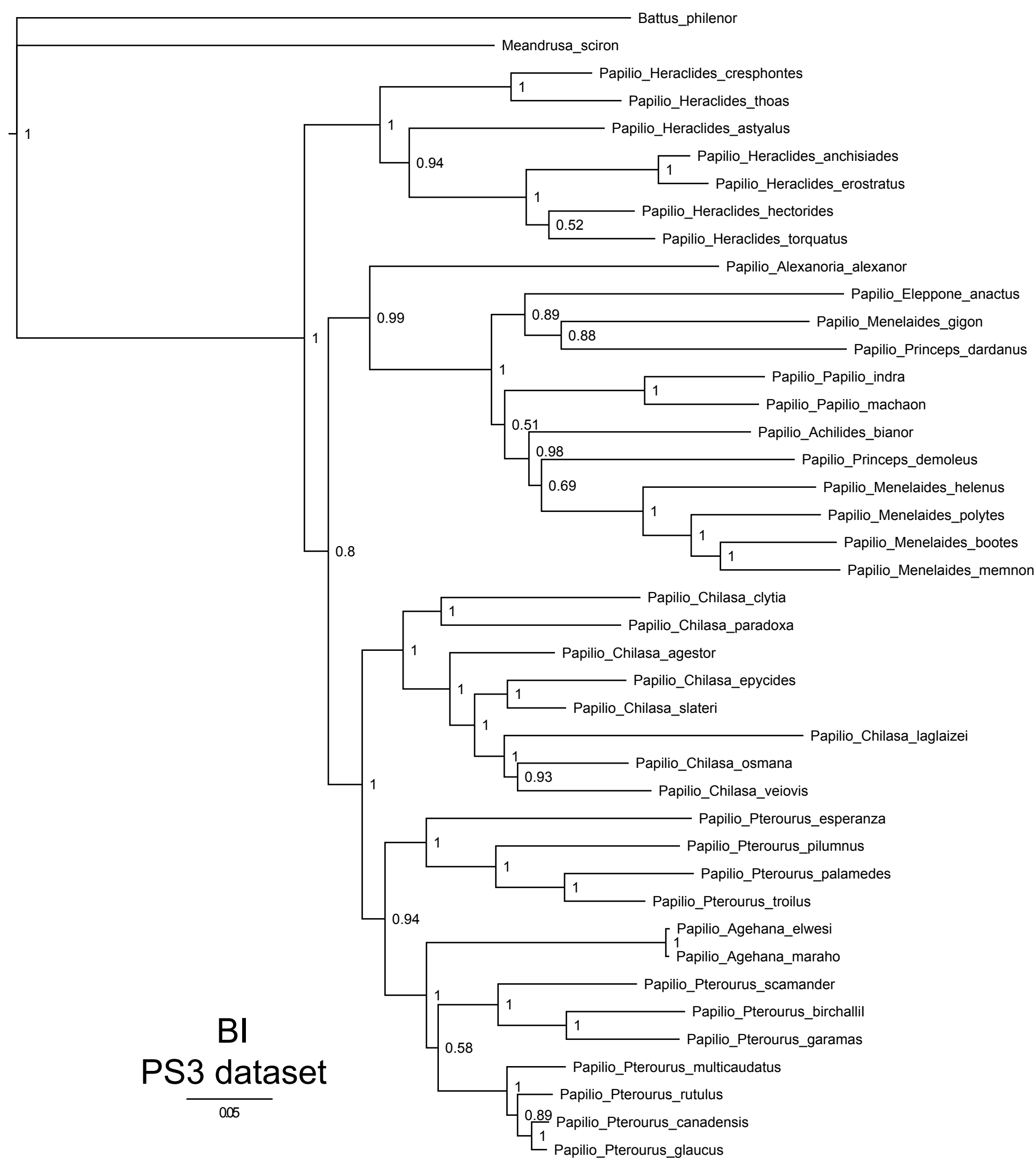

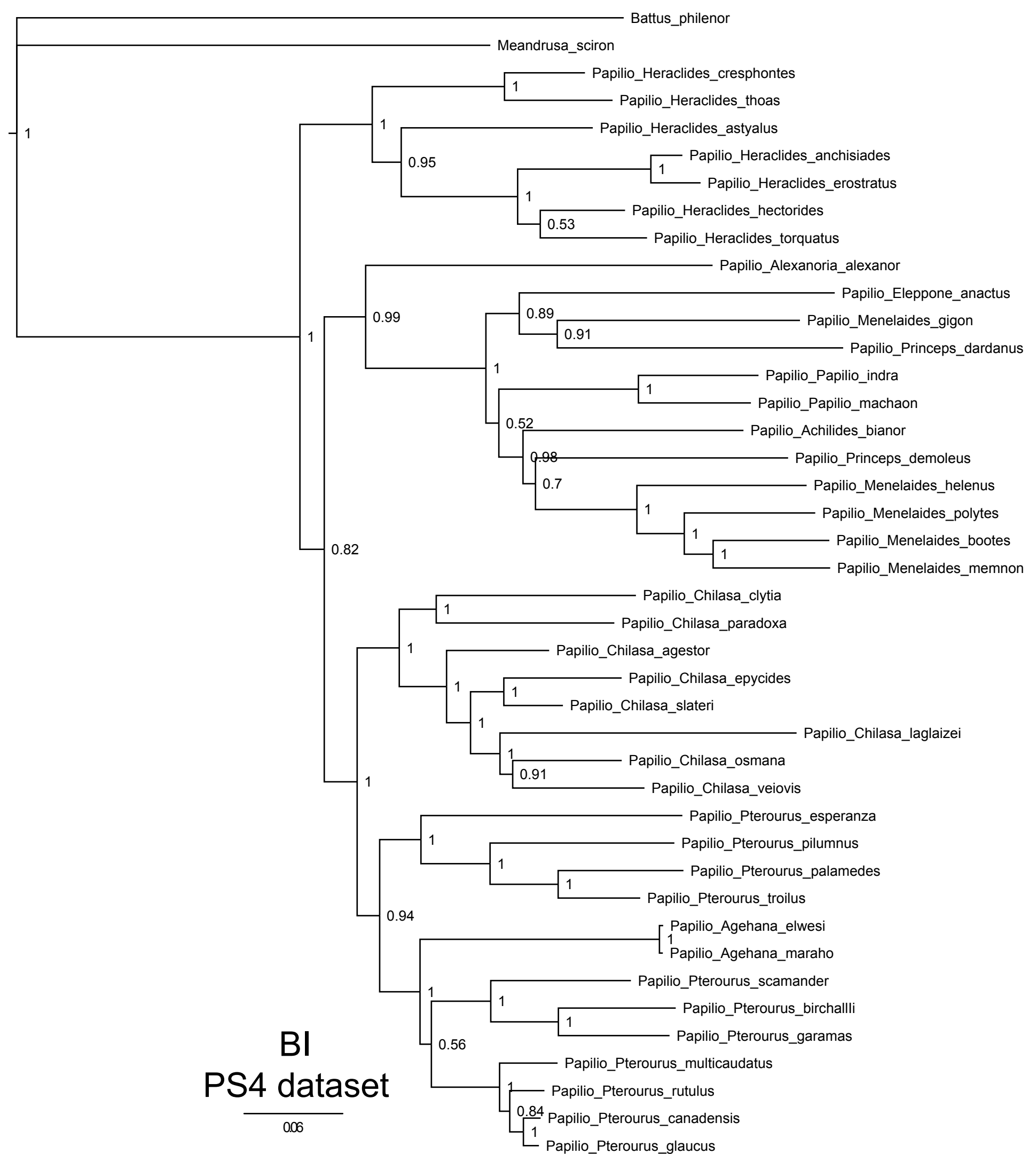

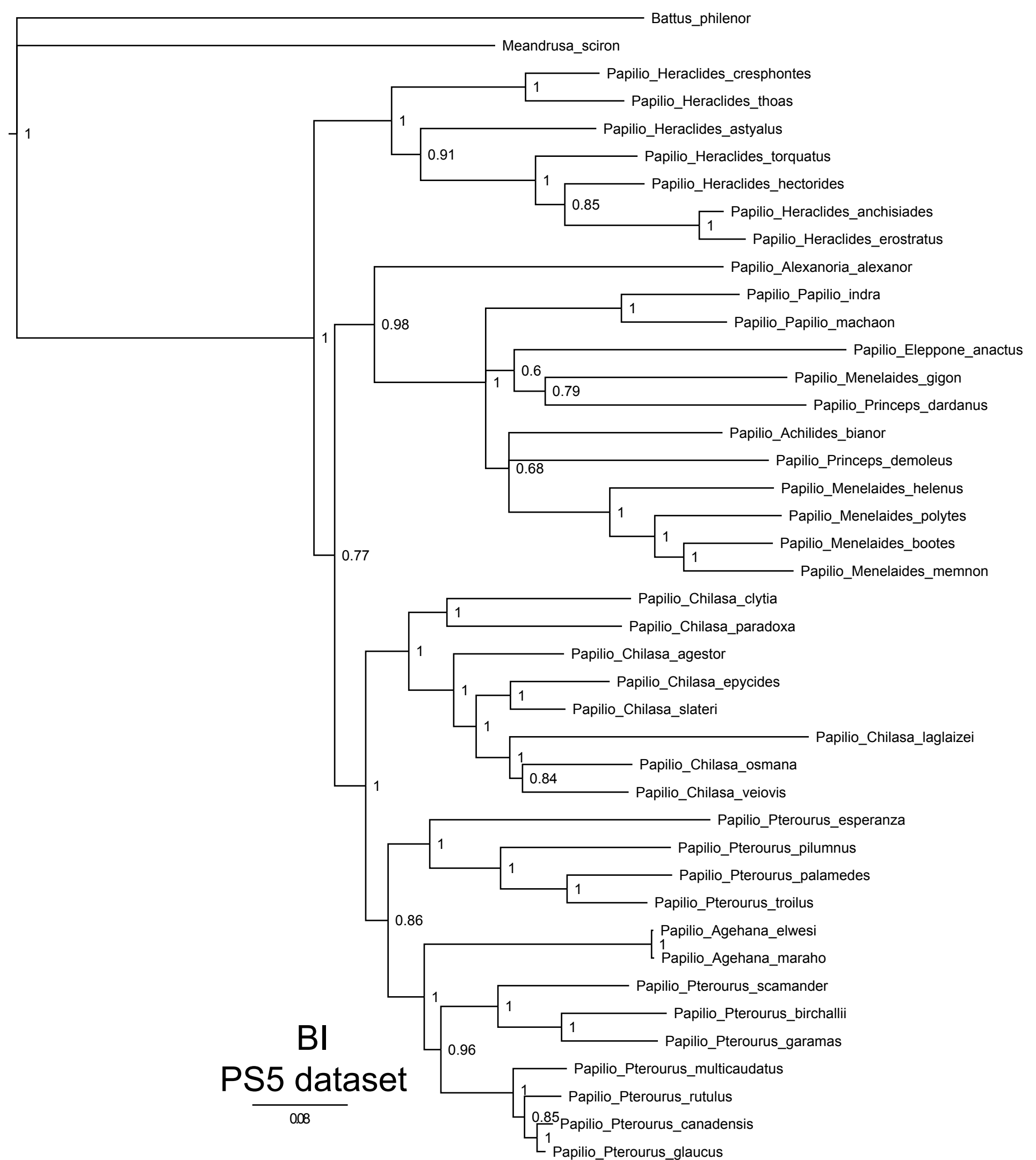

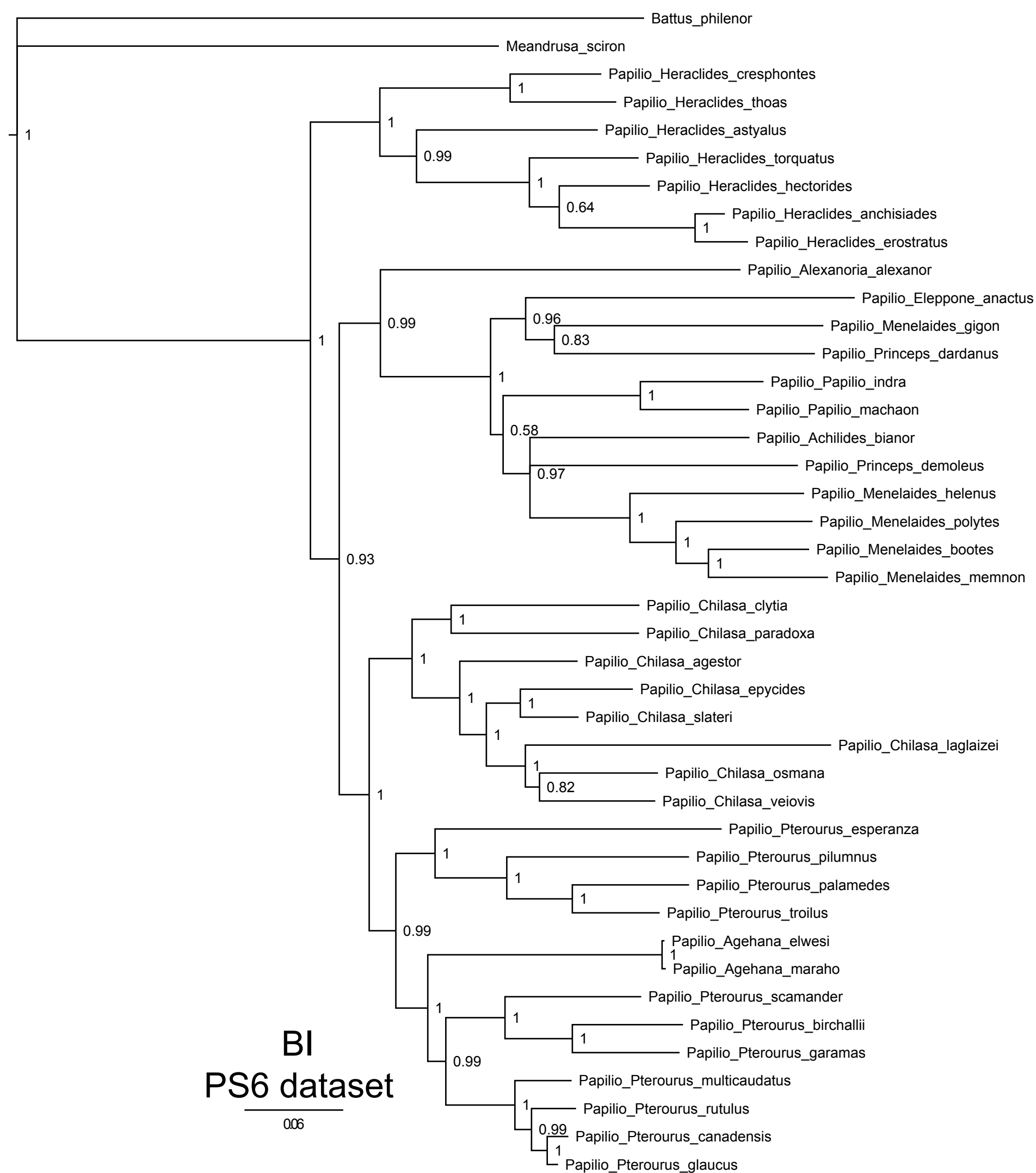

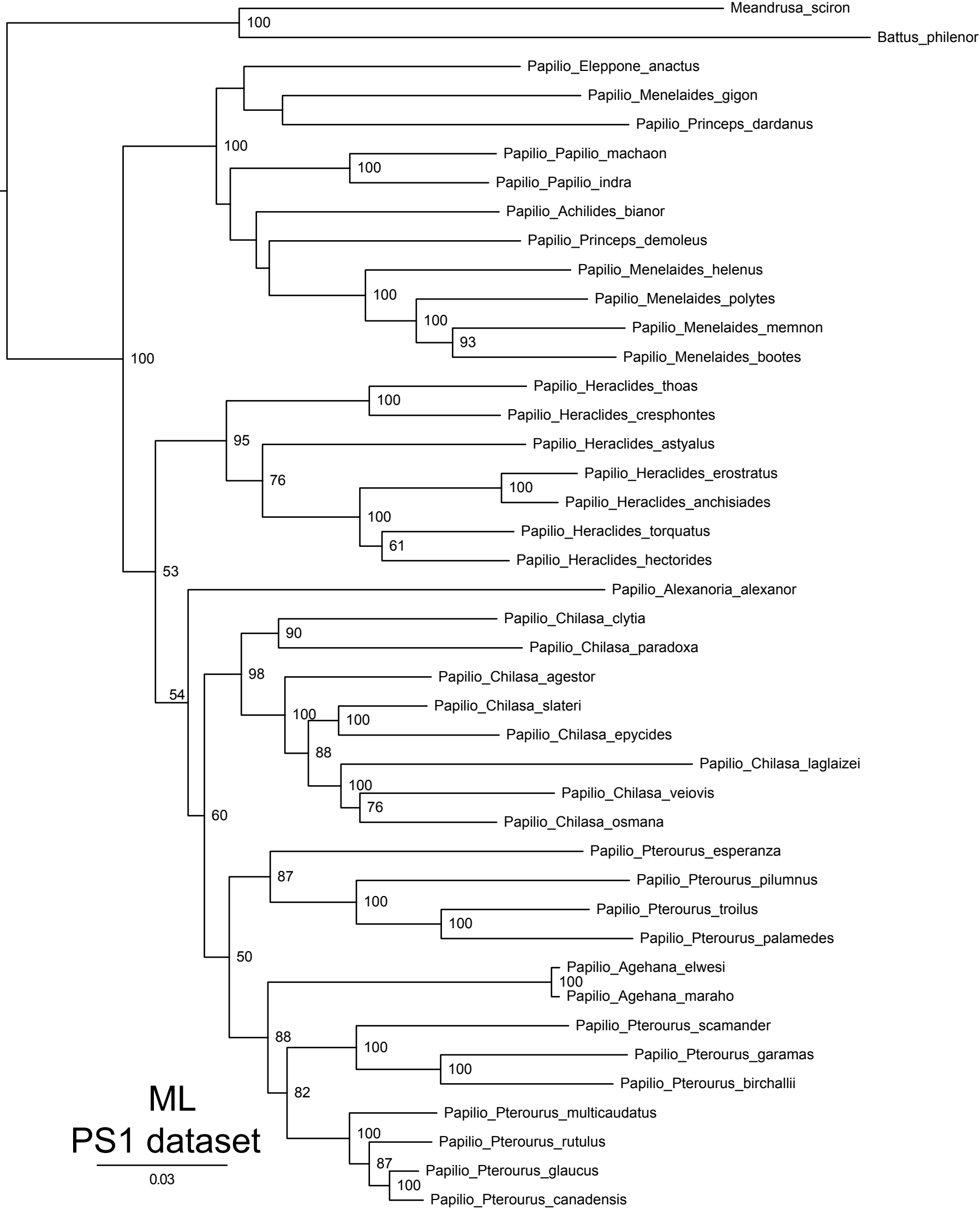

ML  
PS1 dataset  
0.03

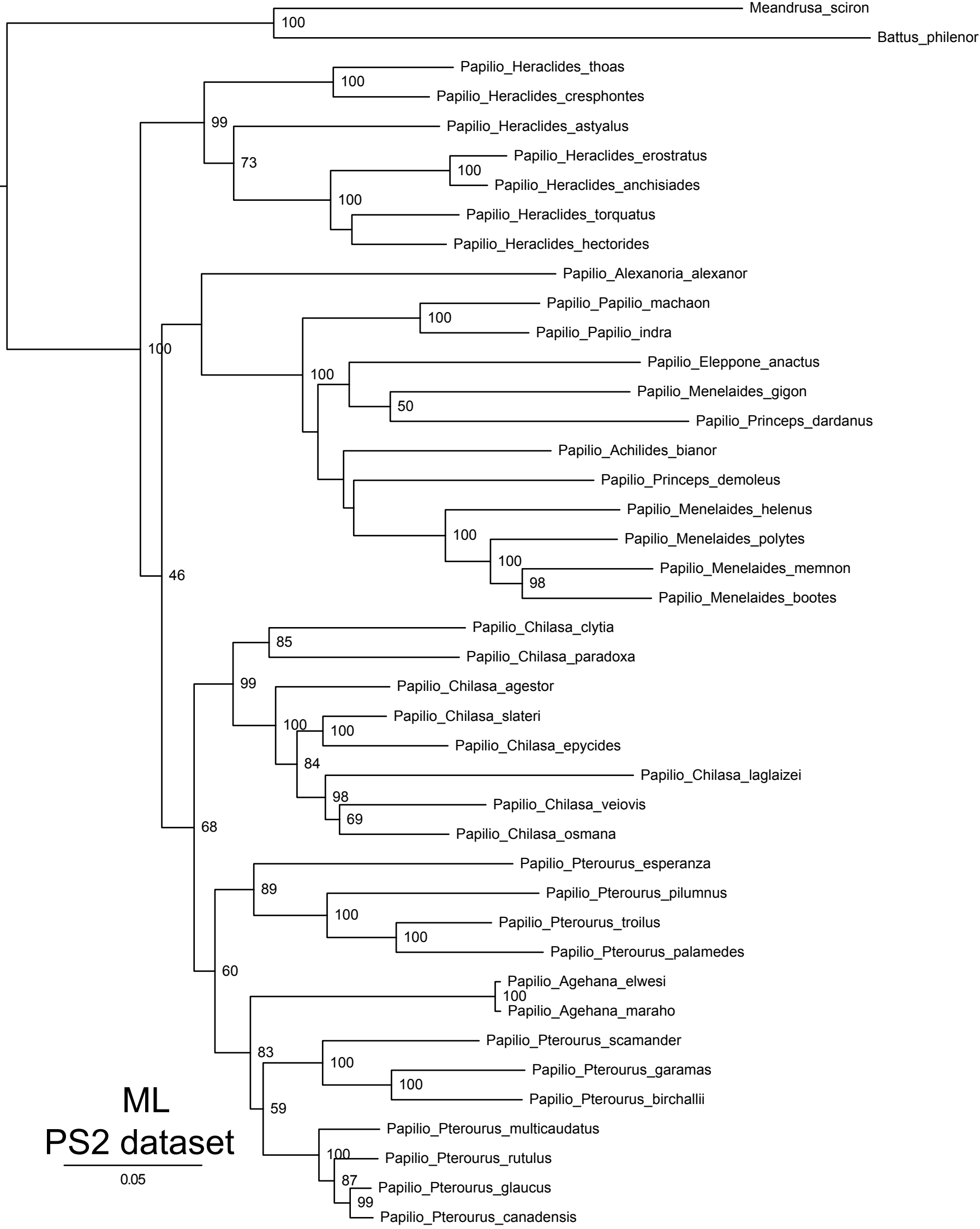

ML  
PS2 dataset  
0.05

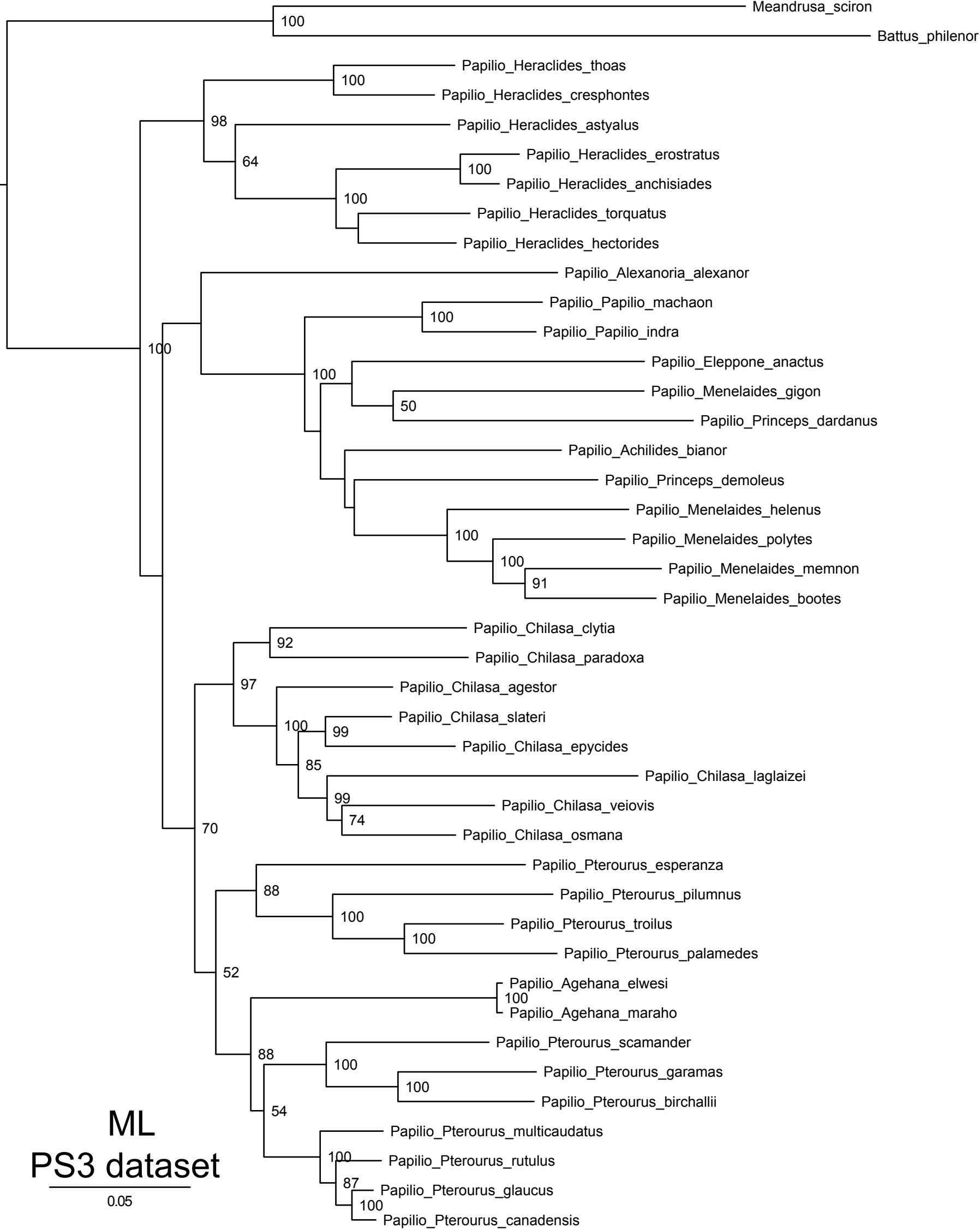

ML  
PS3 dataset  
0.05

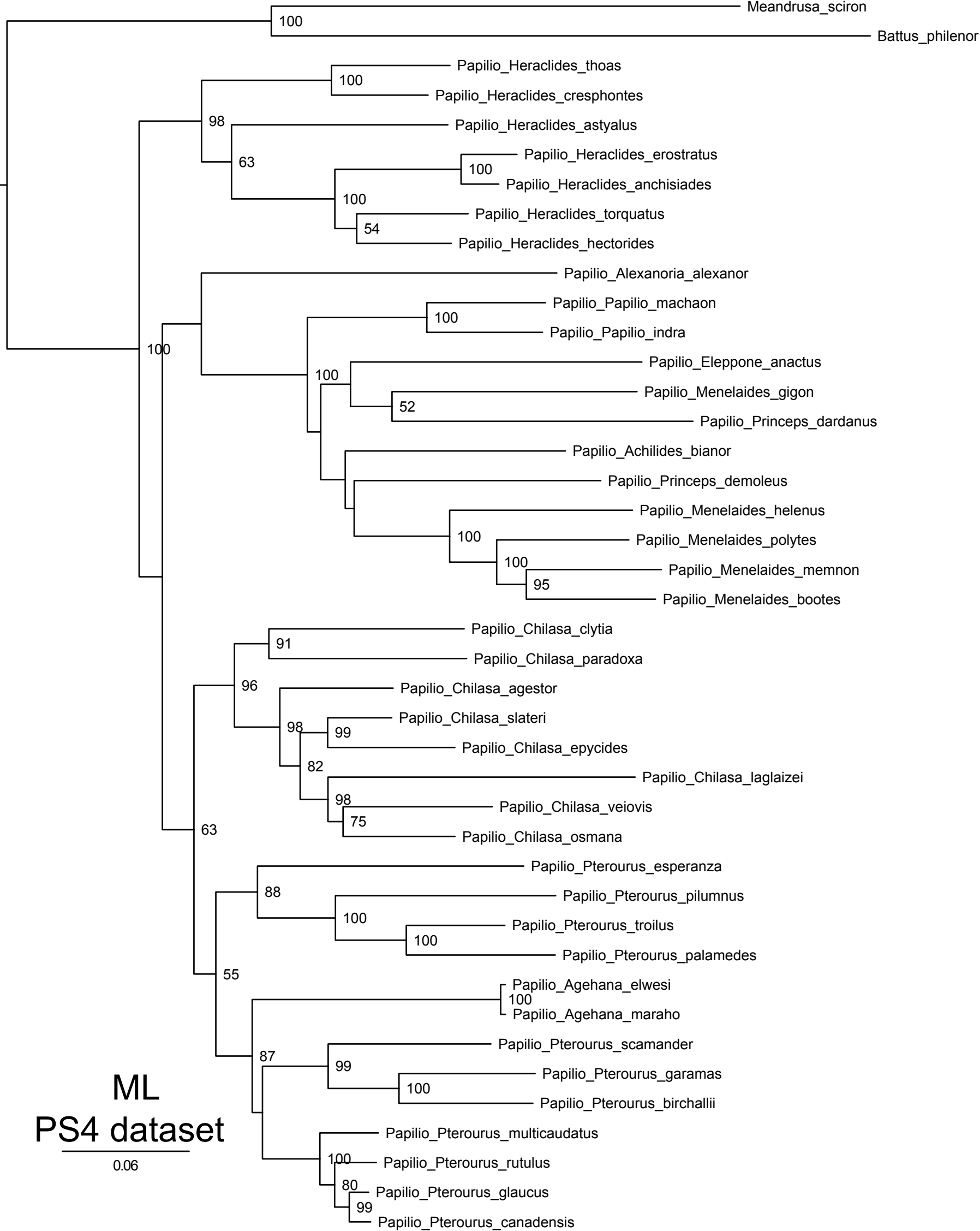

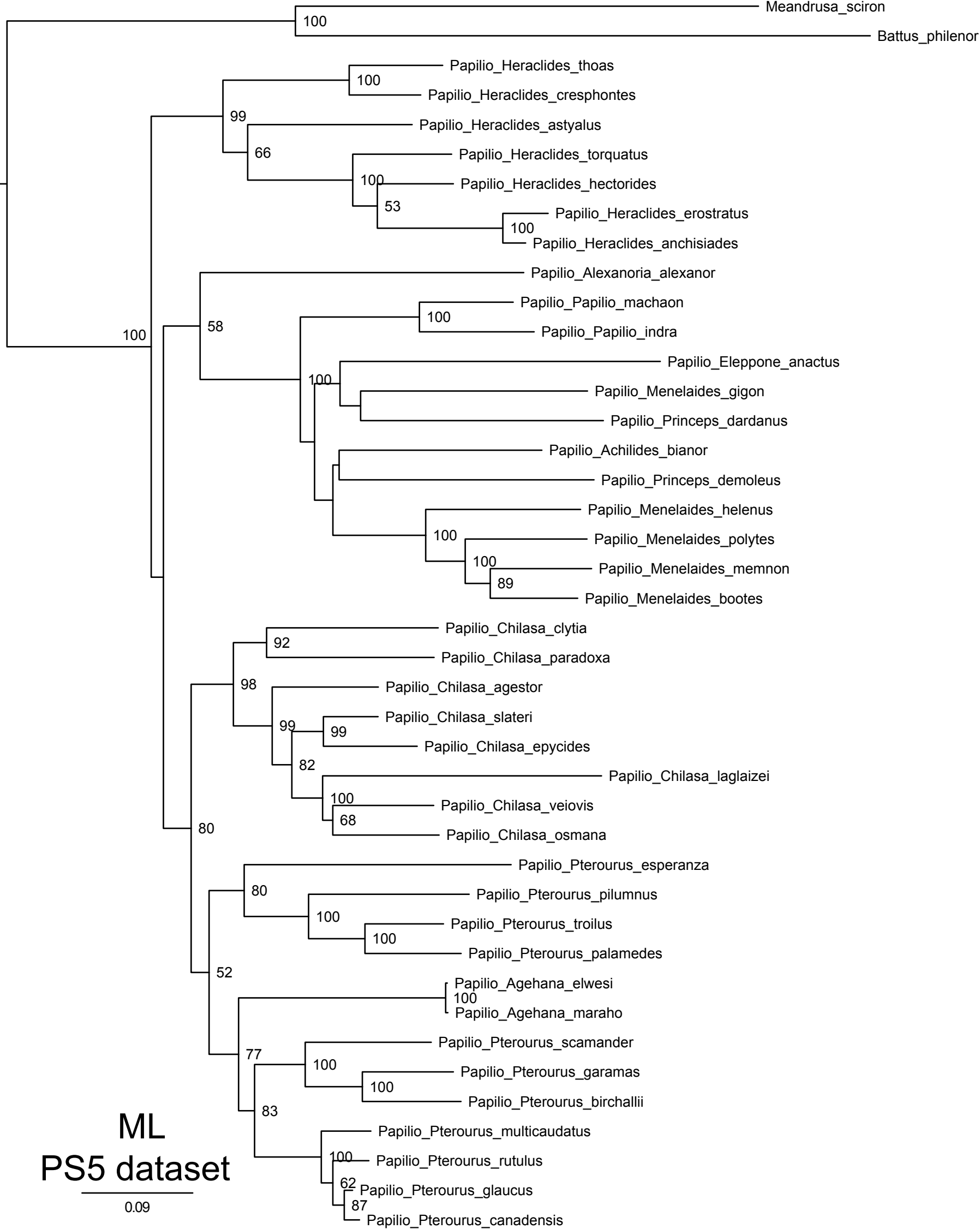

ML  
PS5 dataset  
0.09

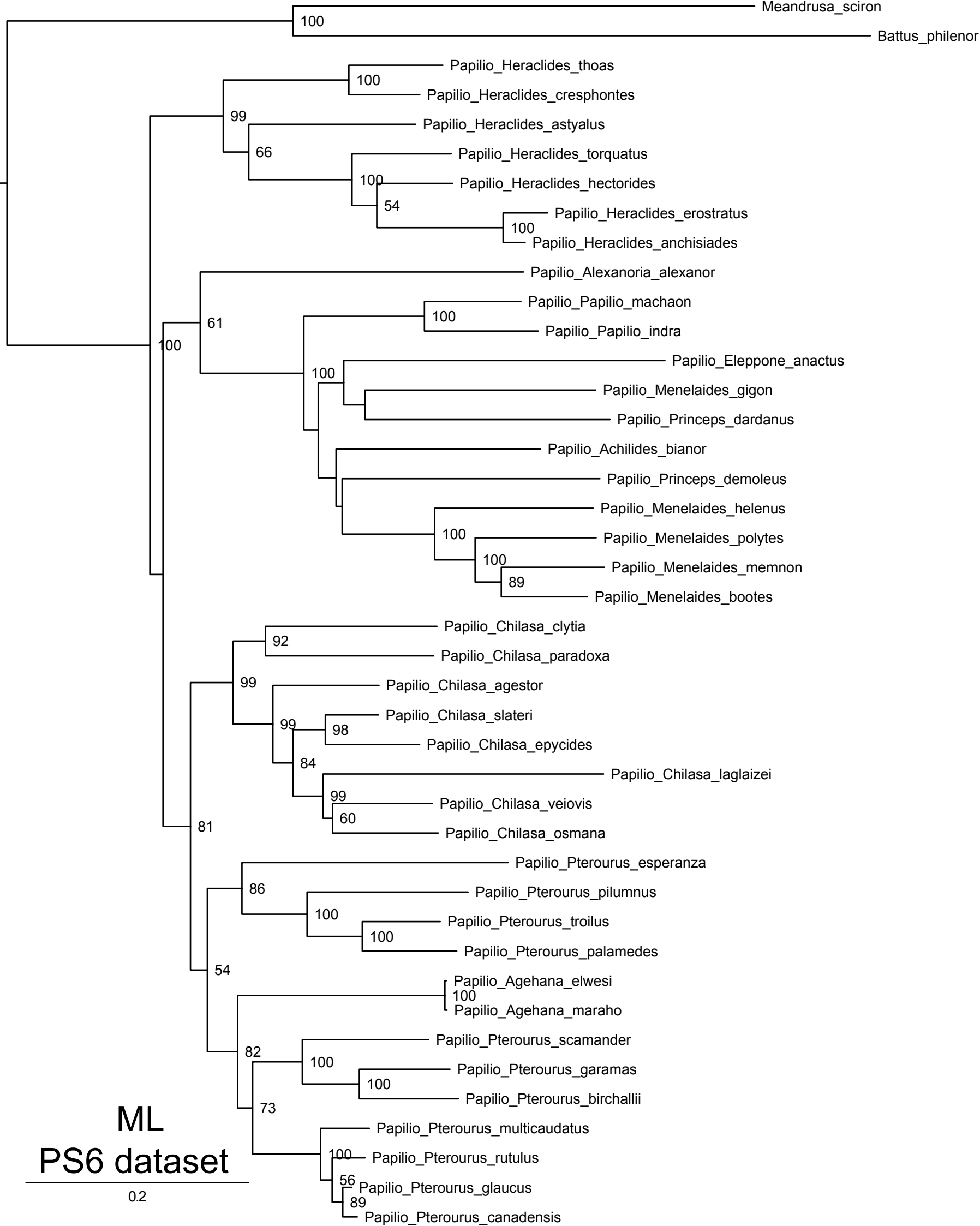

ML  
PS6 dataset  
0.2

Supplement: S3 File — Values at nodes correspond to posterior probabilities or ML bootstrap. (PDF) [file pone.0140933.s005.pdf]
