# Supplementary material for: Phylogeny and Historical Biogeography of Asian Pterourus Butterflies (Lepidoptera: Papilionidae): A Case of Intercontinental Dispersal from North America to East Asia
Source: PLoS One. 2015 Oct 20;10(10):e0140933. doi: 10.1371/journal.pone.0140933 (PMC4617649; doi:10.1371/journal.pone.0140933)
Supplement: S2 File — (PDF) [file pone.0140933.s004.pdf]

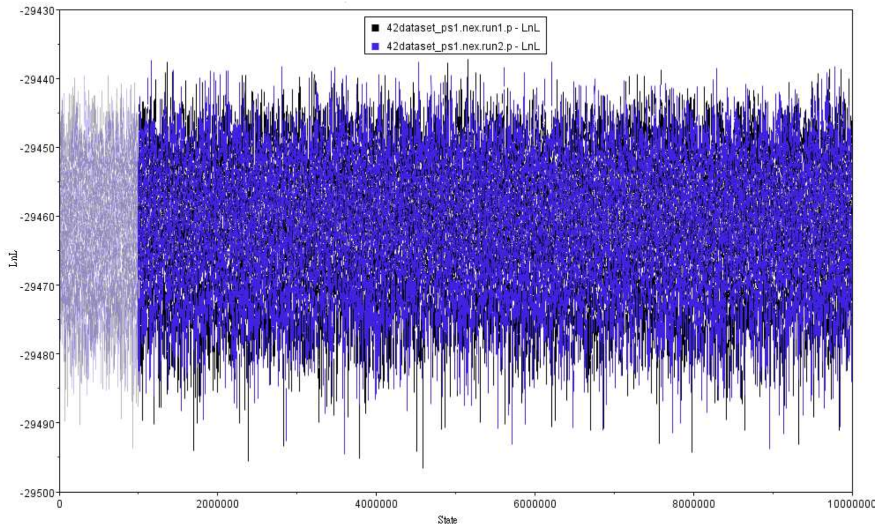

PS1: (A) Trace plot of ln L in Tracer 1.6.

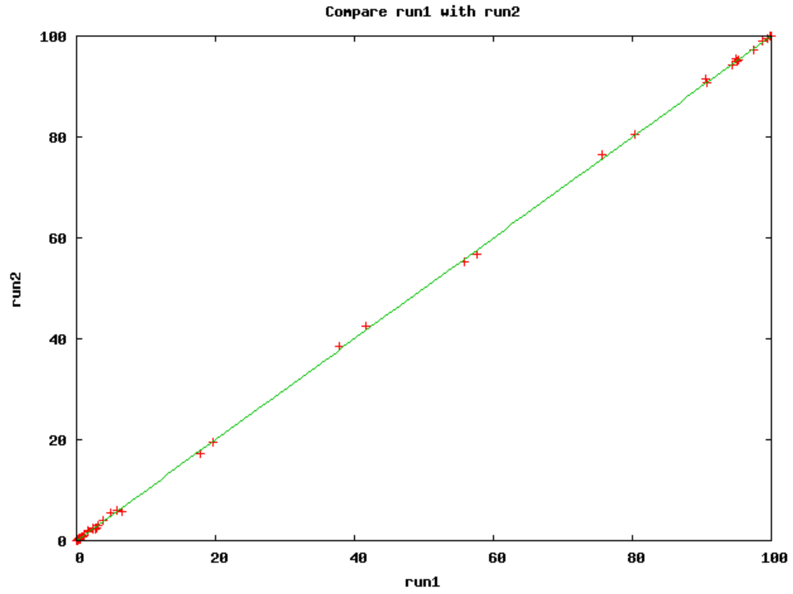

PS1: (B) Simulation run1 compare to run2 in AWTY.

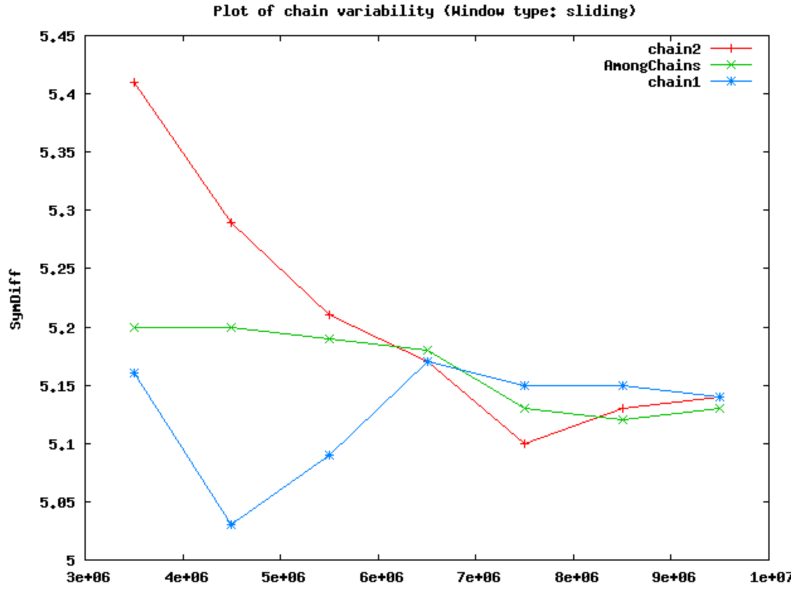

PS1: (C) Tree-distance variation between two runs in AWTY.

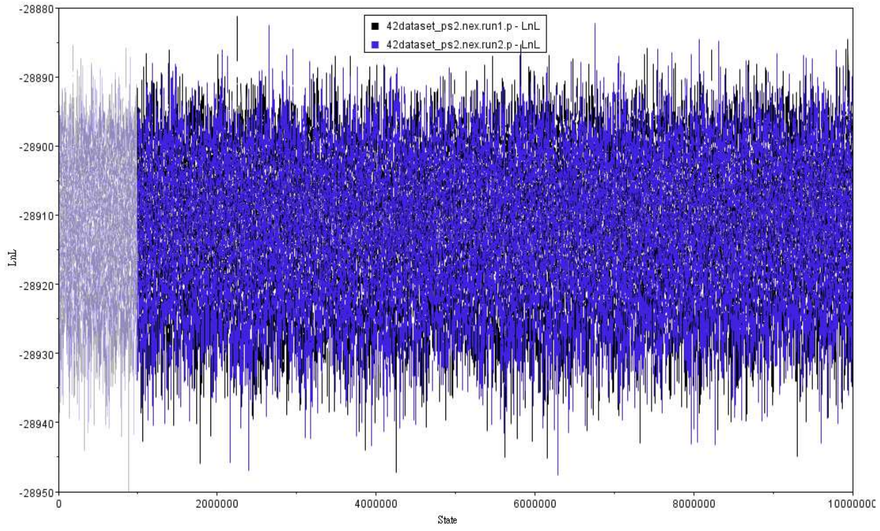

PS2: (A) Trace plot of ln L in Tracer 1.6.

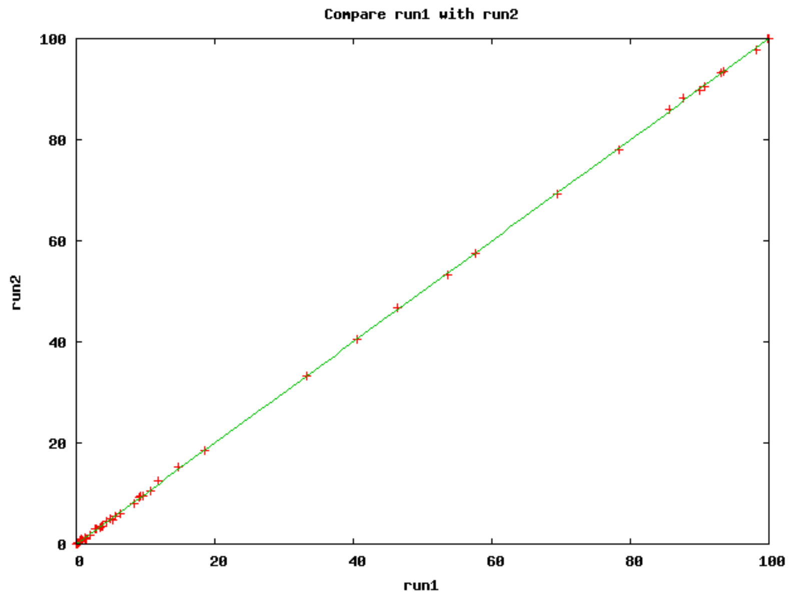

PS2: (B) Simulation run1 compare to run2 in AWTY.

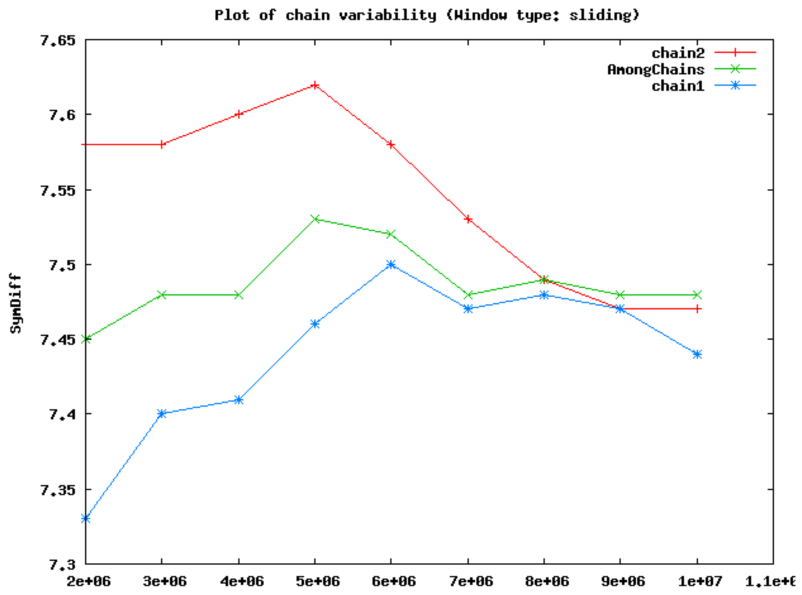

PS2: (C) Tree-distance variation between two runs in AWTY.

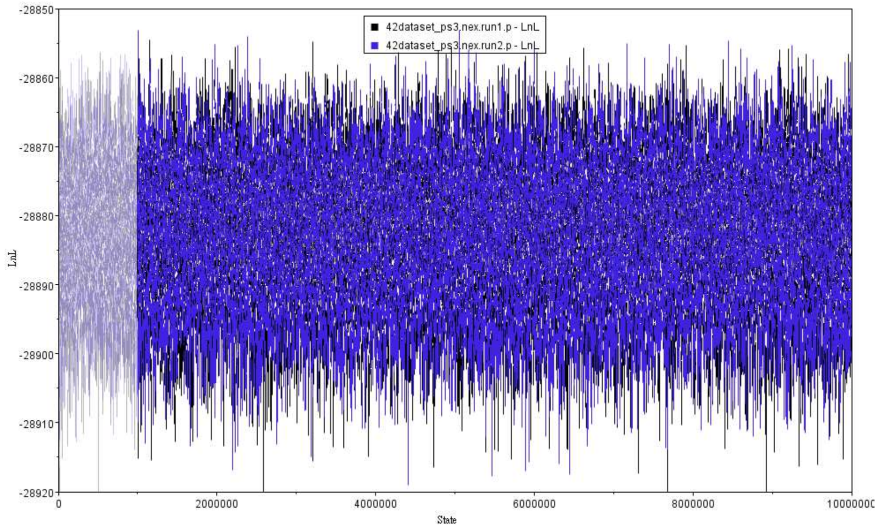

PS3: (A) Trace plot of ln L in Tracer 1.6.

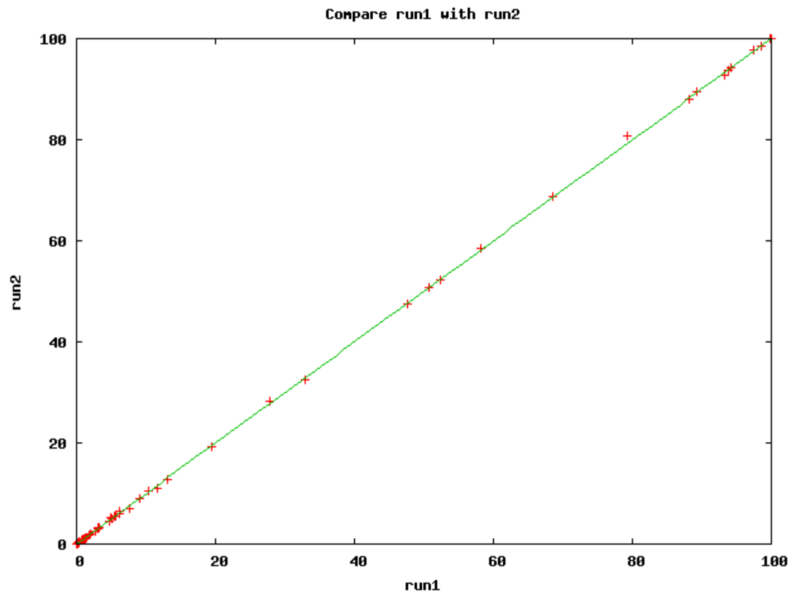

PS3: (B) Simulation run1 compare to run2 in AWTY.

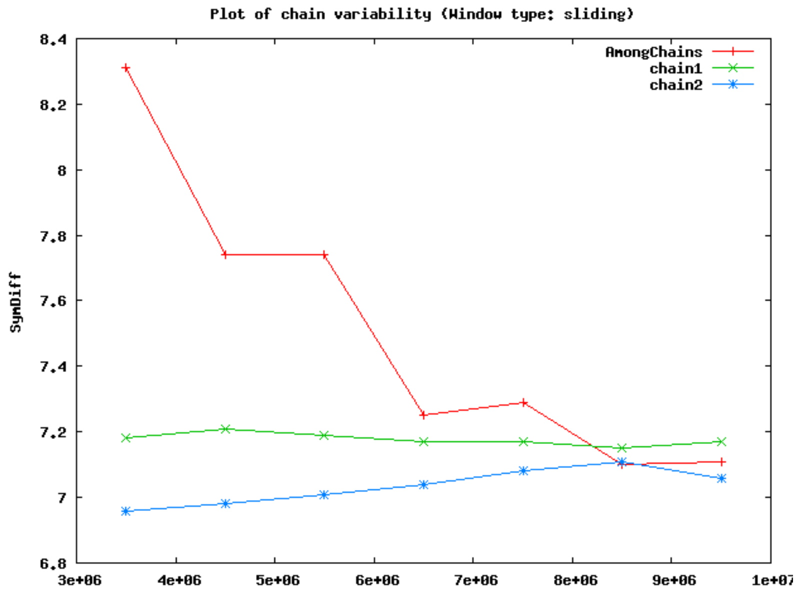

PS3: (C) Tree-distance variation between two runs in AWTY.

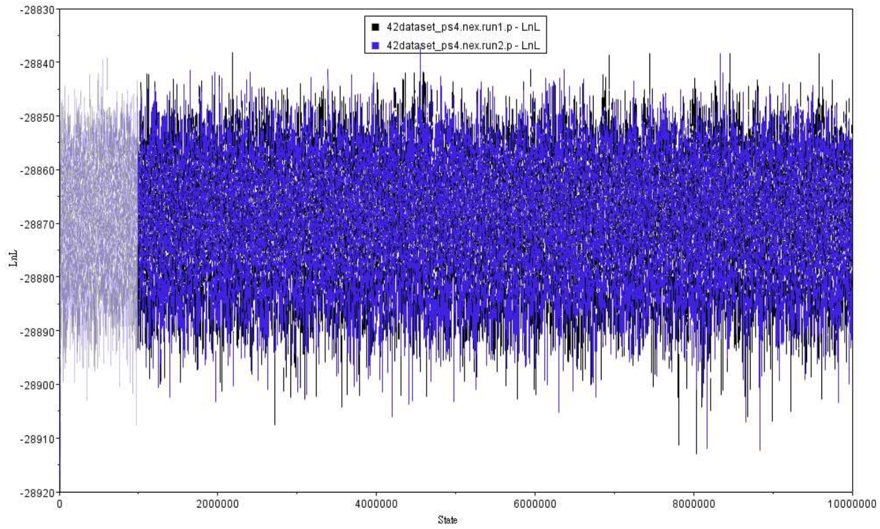

PS4: (A) Trace plot of ln L in Tracer 1.6.

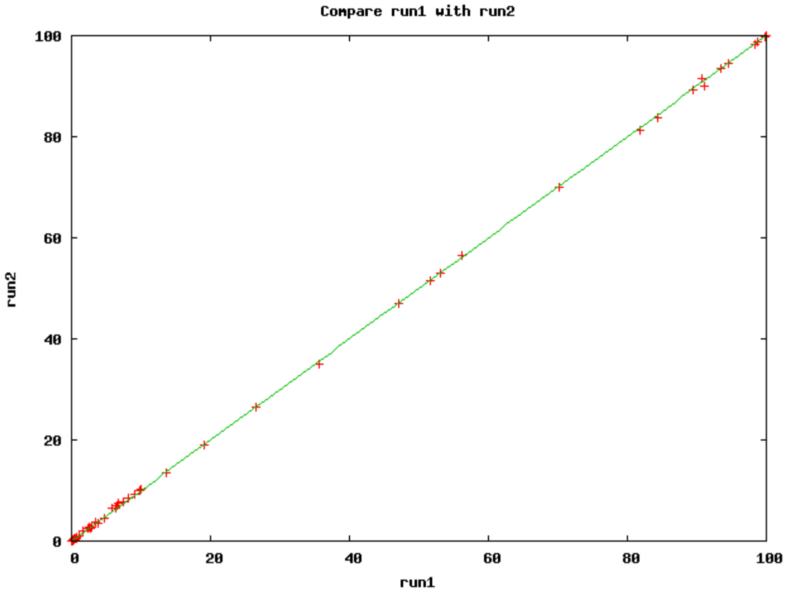

PS4: (B) Simulation run1 compare to run2 in AWTY.

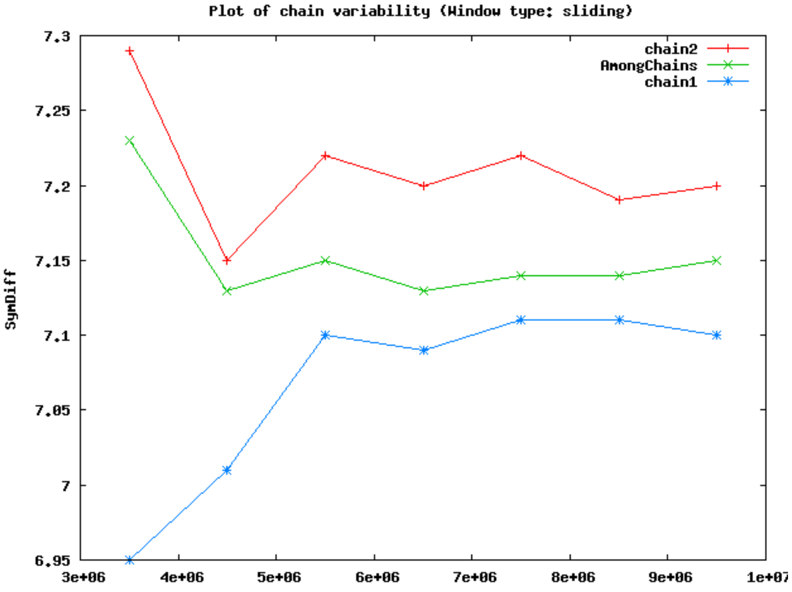

PS4: (C) Tree-distance variation between two runs in AWTY.

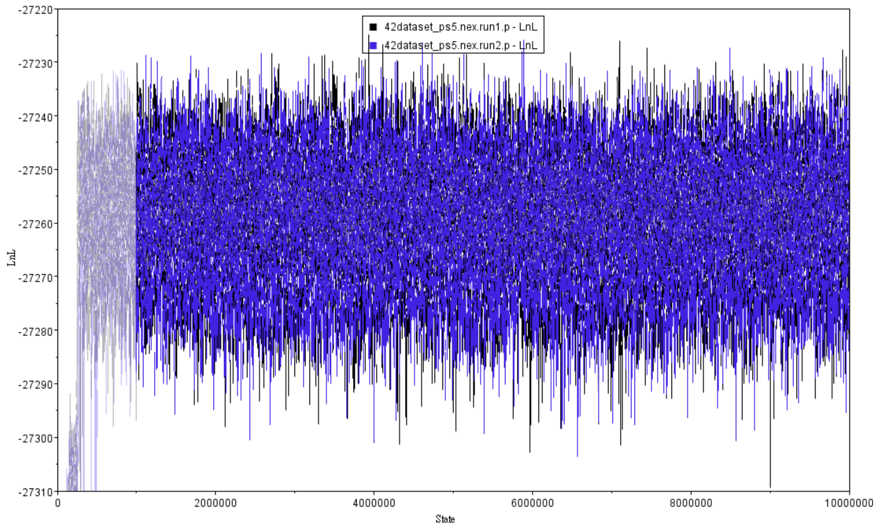

PS5: (A) Trace plot of ln L in Tracer 1.6.

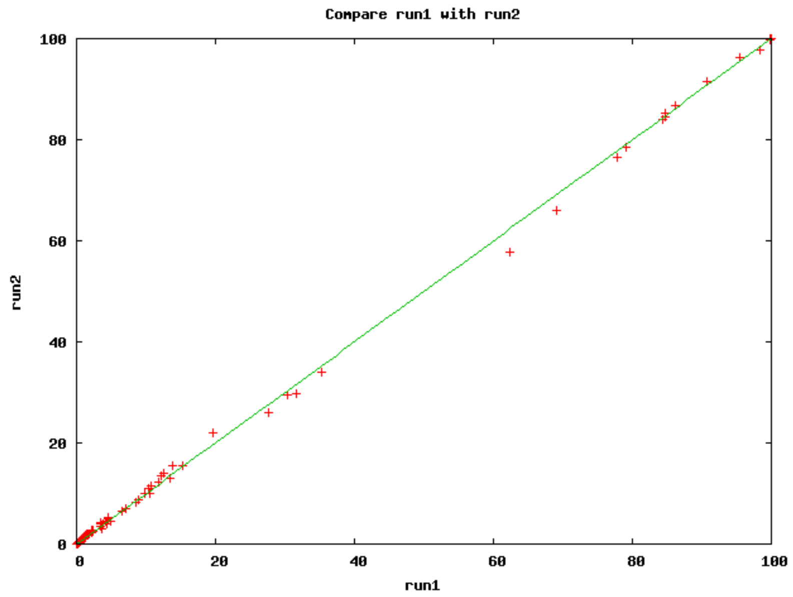

PS5: (B) Simulation run1 compare to run2 in AWTY.

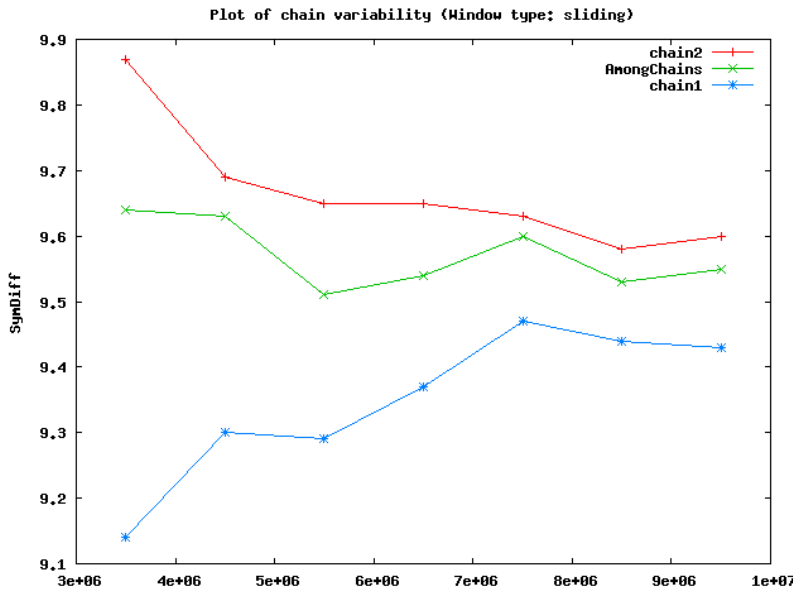

PS5: (C) Tree-distance variation between two runs in AWTY.

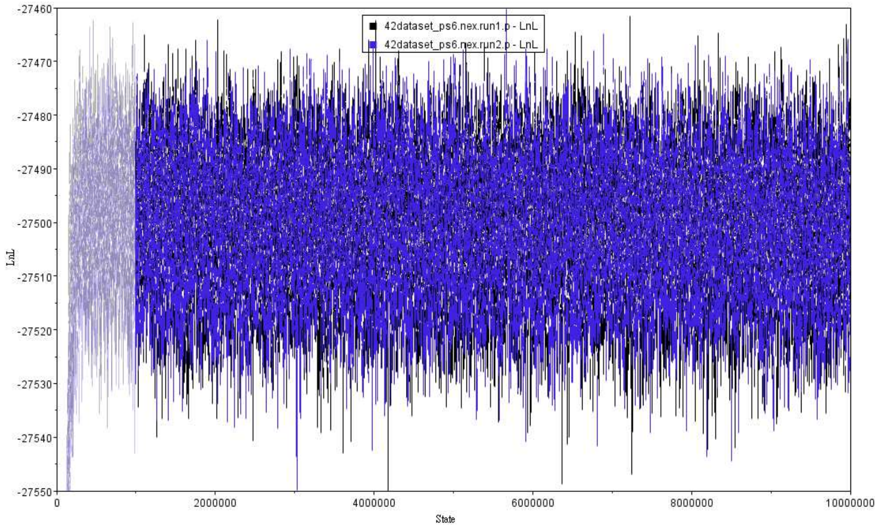

PS6: (A) Trace plot of ln L in Tracer 1.6.

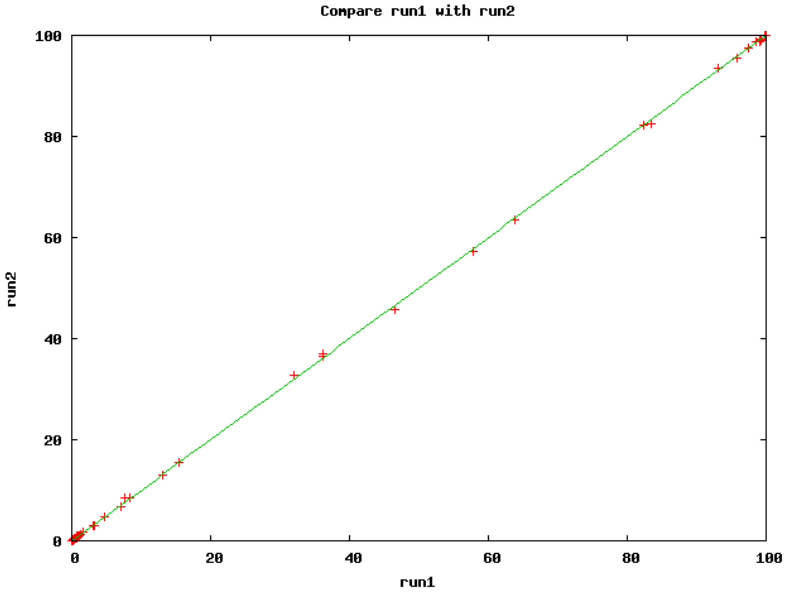

PS6: (B) Simulation run1 compare to run2 in AWTY.

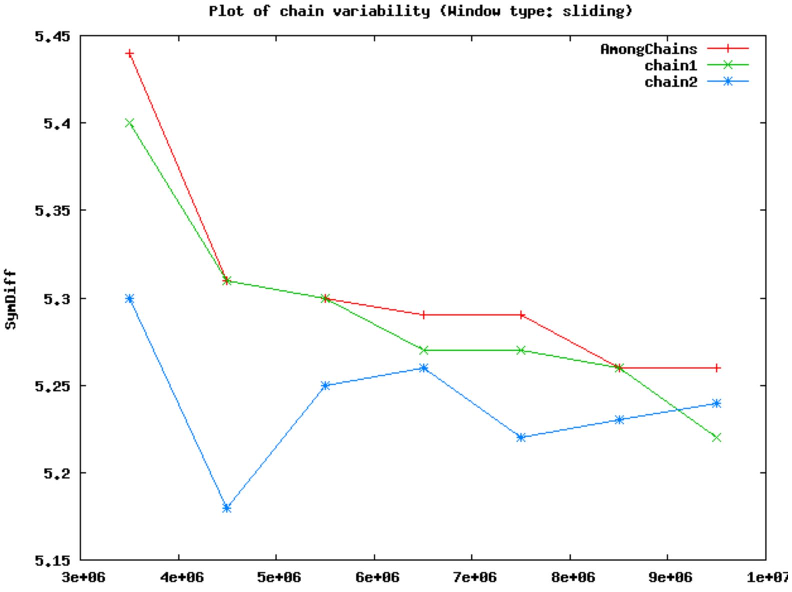

PS6: (C) Tree-distance variation between two runs in AWTY.

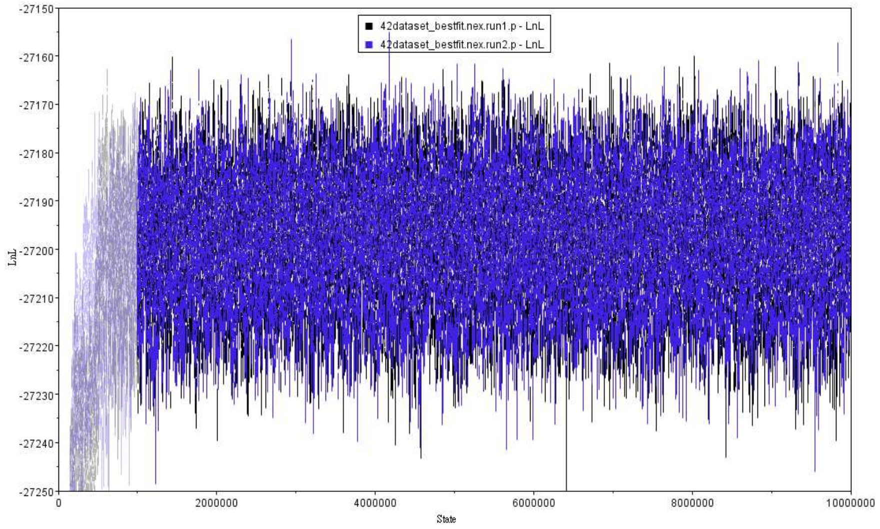

Best-fit PS: (A) Trace plot of ln L in Tracer 1.6.

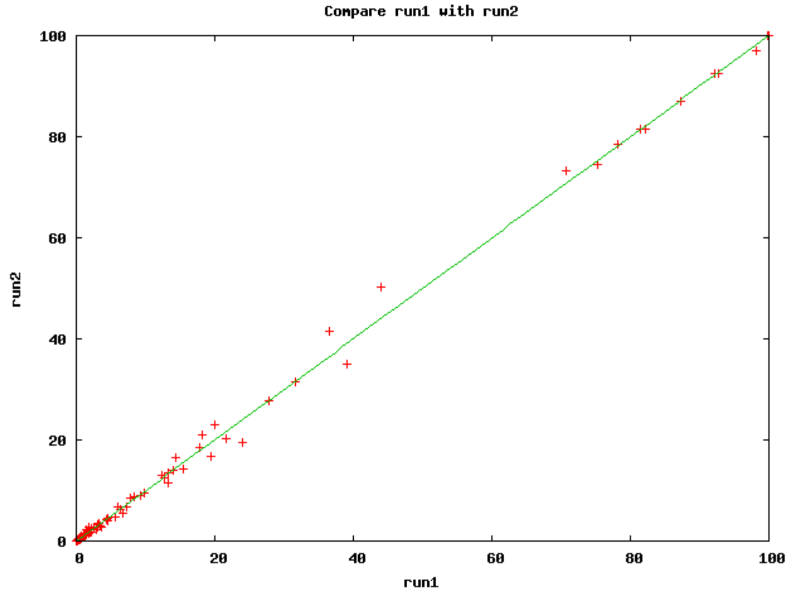

Best-fit PS: (B) Simulation run1 compare to run2 in AWTY.

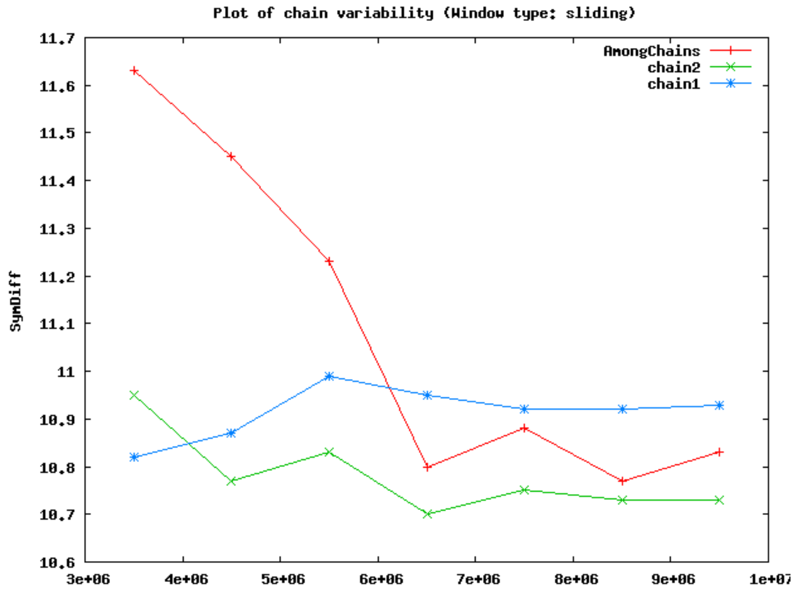

Best-fit PS: (C) Tree-distance variation between two runs in AWTY.
